# ZYG-9^ch-TOG^ promotes the stability of acentrosomal poles via regulation of spindle microtubules in *C*. *elegans* oocyte meiosis

**DOI:** 10.1371/journal.pgen.1010489

**Published:** 2022-11-30

**Authors:** Gabriel Cavin-Meza, Timothy J. Mullen, Emily R. Czajkowski, Ian D. Wolff, Nikita S. Divekar, Justin D. Finkle, Sarah M. Wignall

**Affiliations:** Department of Molecular Biosciences, Northwestern University, Evanston, Illinois, United States of America; University of California Santa Cruz, UNITED STATES

## Abstract

During mitosis, centrosomes serve as microtubule organizing centers that guide the formation of a bipolar spindle. However, oocytes of many species lack centrosomes; how meiotic spindles establish and maintain these acentrosomal poles remains poorly understood. Here, we show that the microtubule polymerase ZYG-9^ch-TOG^ is required to maintain acentrosomal pole integrity in *C*. *elegans* oocyte meiosis. We exploited the auxin inducible degradation system to remove ZYG-9 from pre-formed spindles within minutes; this caused the poles to split apart and an unstable multipolar structure to form. Depletion of TAC-1, a protein known to interact with ZYG-9 in mitosis, caused loss of proper ZYG-9 localization and similar spindle phenotypes, further demonstrating that ZYG-9 is required for pole integrity. However, depletion of ZYG-9 or TAC-1 surprisingly did not affect the assembly or stability of monopolar spindles, suggesting that these proteins are not required for acentrosomal pole structure *per se*. Moreover, fluorescence recovery after photobleaching (FRAP) revealed that ZYG-9 turns over rapidly at acentrosomal poles, displaying similar turnover dynamics to tubulin itself, suggesting that ZYG-9 does not play a static structural role at poles. Together, these data support a global role for ZYG-9 in regulating the stability of bipolar spindles and demonstrate that the maintenance of acentrosomal poles requires factors beyond those acting to organize the pole structure itself.

## Author summary

The spindle is a dynamic microtubule-based structure that functions to segregate duplicated chromosomes during cell division. In many types of cells, protein-rich structures known as centrosomes organize the football-shaped spindle, with the centrosomes functioning to organize microtubules at the two ends (or “poles”) of the structure. However, in female reproductive cells (oocytes) of many organisms, a bipolar spindle is formed and stabilized without centrosomes. Here, we utilized the model organism *C*. *elegans* to understand how various microtubule-associated proteins facilitate the formation and maintenance of these ‘acentrosomal’ poles. We investigated two proteins in *C*. *elegans* oocytes, ZYG-9 and TAC-1, and found that rapid removal of these proteins from the spindle caused the poles to split apart, implicating these factors in stabilizing the acentrosomal pole structure. However, follow-up experiments suggested that ZYG-9 and TAC-1 do not function solely at the poles. Instead, they appear to act more globally to stabilize the entire spindle. Taken together, our findings have expanded the mechanistic model of how spindle bipolarity can be achieved in the absence of centrosomes and pave the way for further studies of acentrosomal pole protein dynamics and functions.

## Introduction

When a cell divides, the genetic material must be accurately partitioned to ensure the viability of the daughter cells. This process is mediated by a bipolar microtubule-based spindle that provides the forces to segregate the chromosomes. In mitotically-dividing cells, two centriole-containing centrosomes nucleate microtubules and organize their minus ends to form the spindle poles, thus providing structural cues that impart stability to the spindle. Conversely, meiotically-dividing oocytes of many organisms lack centrosomes and therefore use distinct mechanisms to focus microtubule minus ends and arrange the spindle poles. Notably, acentrosomal spindle poles in human oocytes are frequently unstable; a significant fraction of oocytes undergo a period of spindle instability in which, following bipolar spindle formation, the poles split apart and come back together multiple times. This instability has adverse consequences, as oocytes that display more pole instability have higher rates of chromosome mis-segregation [1]. However, despite the importance of organizing and stabilizing acentrosomal poles, little is known about what factors promote pole stability in oocytes of any organism.

Here, we use *C*. *elegans* as a model to address the question of how acentrosomal oocyte spindles are stabilized. The spindle assembly pathway has been well documented in this system. Microtubules are first nucleated and the minus ends are sorted outwards away from the chromosomes. The minus ends then organize into multiple nascent poles that coalesce until bipolarity is achieved [2,3]. Importantly, this pathway is similar to what has been described in human oocytes, where multiple poles also form and then coalesce to form a bipolar spindle [1]. A number of factors required for acentrosomal spindle assembly and pole formation in *C*. *elegans* have been identified: MEI-1/2^katanin^ promote the generation of short microtubules [4], KLP-15/16^Kinesin-14^ bundle these microtubules [5,6], KLP-18^Kinesin-12^ and MESP-1 sort microtubules to establish bipolarity [3], ASPM-1 binds to microtubule minus ends and functions to focus the spindle poles in conjunction with dynein [7,8], and KLP-7^MCAK^ acts to limit the number of spindle poles and inhibit excess microtubule polymerization [2,9,10]. Rapid inactivation or depletion of MEI-1, KLP-18, or dynein causes defects in spindle structure [8,11,12], demonstrating that these factors are required for spindle maintenance. However, whether any other pole associated-proteins stabilize the spindle to maintain bipolarity is not known.

Now, we have identified another factor, ZYG-9, that is required for the stability of acentrosomal spindles, and we provide insight into how it performs this function. In mitotically-dividing *C*. *elegans* embryos, ZYG-9 promotes the formation of long astral microtubules that aid in proper positioning of the mitotic spindle [13,14]. In other organisms, ZYG-9 homologs (Stu2, Msps, XMAP215, ch-TOG) have also been shown to be essential for mitotic spindle assembly. In budding yeast, Stu2 is required for proper spindle positioning and orientation in addition to proper metaphase chromosome alignment [15]. In *Drosophila melangaster*, depletion of Msps in S2 cells results in shorter mitotic spindles [16]. In *Xenopus laevis* egg extracts, depletion of XMAP215 results in abnormally short spindles or failure of centrosomes to nucleate microtubules [17]. Finally, in human cells, ch-TOG is necessary for spindle pole integrity, as ch-TOG depletion results in multipolar mitotic spindles [18,19]. Collectively, these phenotypes suggest that ZYG-9 family proteins regulate microtubule-based processes during cell division. This idea is also supported by *in vitro* experiments that demonstrate that ZYG-9-family proteins possess microtubule nucleation and polymerization activity [20–24].

In addition to this work on mitosis, prior studies have also implicated ZYG-9 family proteins as being essential for meiotic spindle assembly. Depletion of Msps results in multipolar spindles in *Drosophila* oocytes [25]. Moreover, in *C*. *elegans*, two early studies reported that spindles do not properly form following ZYG-9 depletion [26,27], and a recent paper demonstrated that ZYG-9 is required for spindle pole coalescence [6]. However, whether ZYG-9 is required to maintain acentrosomal spindle stability once the bipolar spindle forms was not addressed in these prior studies.

To address this question, we optimized and employed a degron-based strategy, enabling us to rapidly deplete ZYG-9 from oocytes. Removal of ZYG-9 from stable, bipolar spindles revealed that ZYG-9 is required to maintain the integrity of acentrosomal spindle poles; within minutes of ZYG-9 depletion, acentrosomal poles split apart and an unstable multipolar structure formed. Strikingly, this phenotype is similar to the unstable acentrosomal spindle poles described in human oocytes [1]. However, despite its essential role in stabilizing bipolar spindles, we found that ZYG-9 depletion had no effect on monopolar spindle formation. This demonstrates that ZYG-9 is not required for pole integrity in all contexts; rather, the spindle destabilization seen following ZYG-9 depletion from pre-formed spindles likely arises from global effects on the spindle. Thus, our work has revealed new insight into mechanisms required for preserving meiotic spindle integrity and suggests that proper regulation of spindle microtubules is required to maintain the integrity of acentrosomal poles.

## Results

### A degron-based approach to investigate ZYG-9

**Fig 1:**
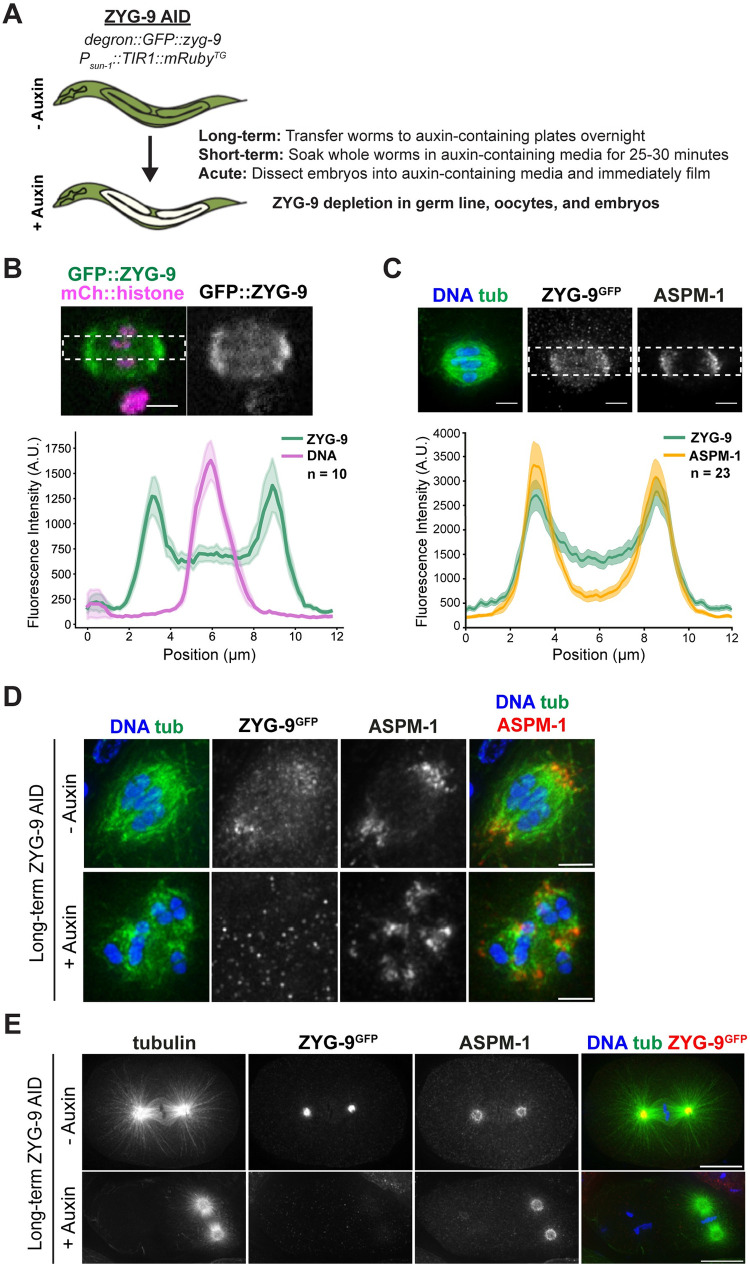
Validation of a ZYG-9-AID strain. (A) Schematic of the degron::GFP::ZYG-9 AID strain and description of auxin treatment lengths. ZYG-9 is tagged at the endogenous locus and the TIR1 transgene is expressed from a germline-specific promoter. Types of auxin treatment: (1) Long-term: worms are incubated on auxin-containing plates overnight, (2) Short-term: worms are soaked in auxin-containing media for 25–30 minutes prior to dissecting oocytes for immunofluorescence, (3) Acute: embryos are dissected into auxin-containing media and filmed immediately. (B) Linescan profiles of degron::GFP::ZYG-9 (green) and mCherry::histone (magenta) fluorescence intensities from metaphase spindles in live oocytes. The plot shows that ZYG-9 is enriched at the poles of the bipolar metaphase spindle, but ZYG-9 is localized in the midspindle region as well. n represents the number of spindles analyzed. Bar = 2.5 μm. (C) Linescan profiles of fixed metaphase spindles stained for DNA (blue), tubulin (green), degron::GFP::ZYG-9 (using the GFP antibody), and ASPM-1. The plot shows that ZYG-9 is enriched at the spindle poles but also has more signal in the midspindle region than ASPM-1. n represents the number of spindles analyzed. Bar = 2.5μm. (D) Meiotic oocyte spindles from ZYG-9 AID worms plated overnight on control plates (- auxin) and 1 mM auxin-containing plates (+ auxin) stained for DNA (blue), tubulin (green), ZYG-9 (not shown in merge), and ASPM-1 (red). Long-term ZYG-9 depletion results in the formation of multipolar spindles (44/45 spindles). Bars = 2.5μm. (E) Mitotic spindles in 1-cell stage embryos from ZYG-9 AID worms plated overnight on control plates (- auxin) and 1 mM auxin-containing plates (+ auxin) stained for DNA (blue), tubulin (green), ZYG-9 (red), and ASPM-1 (not shown in merge). Long-term ZYG-9 depletion recapitulates published mitotic phenotypes (8/8 embryos) [14,33]. Bars = 10μm.

Although spindle poles in human oocytes are often unstable, the mechanisms required to stabilize poles are poorly understood. A previous study found that acentrosomal poles fail to coalesce in *C*. *elegans* oocytes when ZYG-9 is depleted using RNAi, resulting in the formation multipolar rather than bipolar spindles [6]. This finding raised the possibility that ZYG-9 may help stabilize the pole structure, to ensure the formation of two robust poles. We therefore sought to test this hypothesis, by rapidly depleting ZYG-9 from oocytes in which spindles had already established bipolarity, and then assessing effects on the poles. To this end, we took advantage of the auxin-inducible degron (AID) system [28], using CRISPR to introduce a degron::GFP tag at the endogenous locus of *zyg-9* in a worm strain that expresses the TIR1 ubiquitin ligase from a germline-specific promoter (hereafter referred to as “ZYG-9 AID”; Fig 1A). This allows for proteasome-mediated degradation of degron-tagged ZYG-9 upon addition of the small molecule auxin.

To validate this strain, we first assessed whether degron::GFP::ZYG-9 retained its normal localization; live imaging of oocytes demonstrated that tagged ZYG-9 begins to associate with microtubules at the multipolar stage, becoming enriched at the poles and increasing in intensity as the bipolar spindle forms (Figs S1A and 1B and S1 Video, n = 5), as previously reported [6]. Fixed imaging comparing the localization of degron::GFP::ZYG-9 to the spindle pole protein ASPM-1 confirmed these findings (Figs 1C and S1B). Next, we incubated worms on auxin-containing plates overnight to mimic the long-term depletion achieved by RNAi; immunofluorescence (Fig 1D and 1E) and western blotting (S1C Fig) confirmed that we were achieving efficient depletion. Under these conditions, we observed multiple ASPM-1-labeled poles in oocytes (Fig 1D, 44/45 spindles), consistent with ZYG-9’s role in pole focusing [6]. Moreover, examination of mitotic spindles in 1-cell stage embryos after auxin treatment revealed misoriented mitotic spindles with shorter astral microtubules (Fig 1E, 8/8 embryos), which phenocopies the mitotic defects reported for RNAi-mediated ZYG-9 depletion. Finally, we assessed embryonic lethality to determine if addition of the degron::GFP tag had any unintended consequences on ZYG-9 function. Notably, the level of embryonic lethality was similar to wild-type worms when our AID strain was grown in the absence of auxin (0.37% +/- 0.2%), while worms grown on auxin-containing plates exhibited embryonic lethality at levels comparable to those reported in previous studies of *zyg-9* mutants (98.2% +/- 0.6%) [13]. Together, these observations confirmed that our ZYG-9 AID strain was working as expected.

### ZYG-9 is required to maintain acentrosomal spindle pole integrity

**Fig 2:**
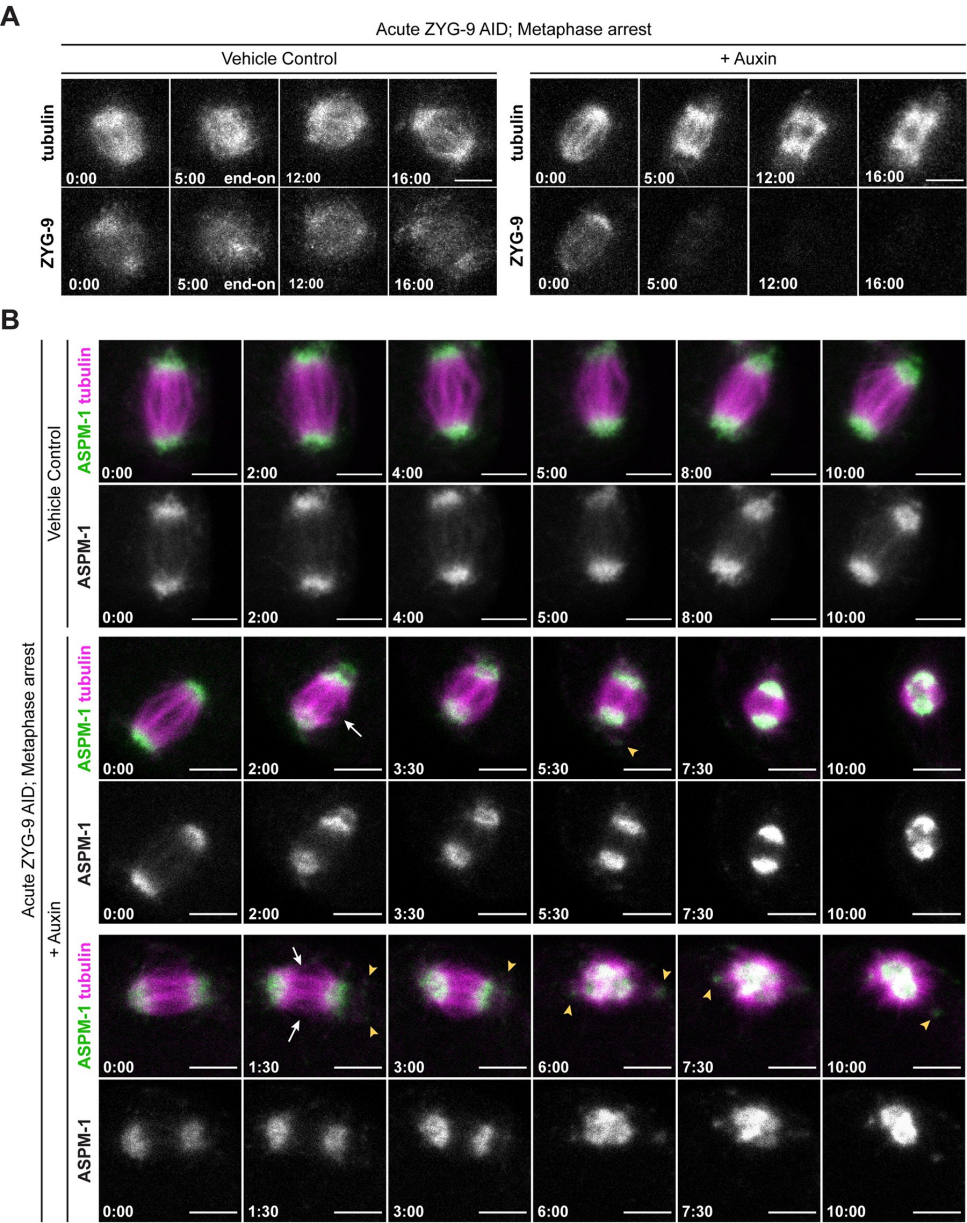
ZYG-9 is required to maintain spindle pole integrity. (A) Movie stills from Metaphase I-arrested *(emb-30(RNAi))* oocytes expressing mCherry::tubulin and degron::GFP::ZYG-9 acutely treated with either vehicle (left) or 100μM auxin (right). Following vehicle treatment, the oocyte spindle maintains ZYG-9 on the spindle and remains bipolar throughout the timecourse (n = 4). Auxin treatment causes a rapid loss of ZYG-9 signal and a concurrent loss of spindle pole stability highlighted by the unfocusing and fragmentation of the spindle poles (n = 4). Bars = 5 μm. Timestamp = min:sec. (B) Movie stills from Metaphase I-arrested *(emb-30(RNAi))* oocytes expressing mCherry::tubulin, degron::GFP::ZYG-9, and GFP::ASPM-1 to track acentrosomal poles. Stable bipolar spindles have clear, focused ASPM-1 foci on either side of the spindle in control oocytes (n = 4). Acute 100μM auxin treatment to deplete ZYG-9 causes a rapid unfocusing and fragmentation of ASPM-1 foci, confirming loss of spindle bipolarity (n = 5). Splayed microtubule bundles can be seen in the midspindle region (white arrows) and fragments of ASPM-1 can be seen dissociating from poles (yellow arrowheads). Note that the spindle in rows 5 and 6 rotates to an end-on orientation by the end of the timelapse. Bars = 5 μm. Timestamp = min:sec.

After validating our ZYG-9 AID strain, we next attempted to acutely deplete ZYG-9 and assess the stability of oocyte spindle poles that had already formed. To this end, we arrested oocytes at Metaphase I through RNAi-mediated depletion of EMB-30, a component of the anaphase promoting complex [29], in a strain containing both degron::GFP::ZYG-9 and mCherry::tubulin. We then dissected these arrested oocytes into auxin-containing media and immediately mounted them for live imaging. Upon acute auxin treatment, we observed a rapid loss in GFP::ZYG-9 fluorescence within minutes, demonstrating that ZYG-9 depletion was occurring. Strikingly, as ZYG-9 was being depleted, the spindle poles began to split apart and fragment (Fig 2A and S2 and S3 Videos, n = 4). In contrast, oocytes dissected into media without auxin maintained bipolarity over the entire time course (Fig 2A and S4 Video, n = 4). To confirm that poles were fragmenting, we repeated these experiments in a ZYG-9 AID strain expressing GFP::ASPM-1 and mCherry::tubulin. In control metaphase-arrested oocytes, two prominent ASPM-1 foci were maintained throughout the duration of imaging, demonstrating that bipolarity was maintained (Fig 2B, rows 1 and 2, S5 Video, n = 4). In contrast, ASPM-1 foci began to split and fragment upon dissection of oocytes into auxin (Fig 2B, rows 3–6 yellow arrows, S6 and S7 Videos, n = 5). These findings demonstrate that ZYG-9 is required to maintain acentrosomal pole stability.

**Fig 3:**
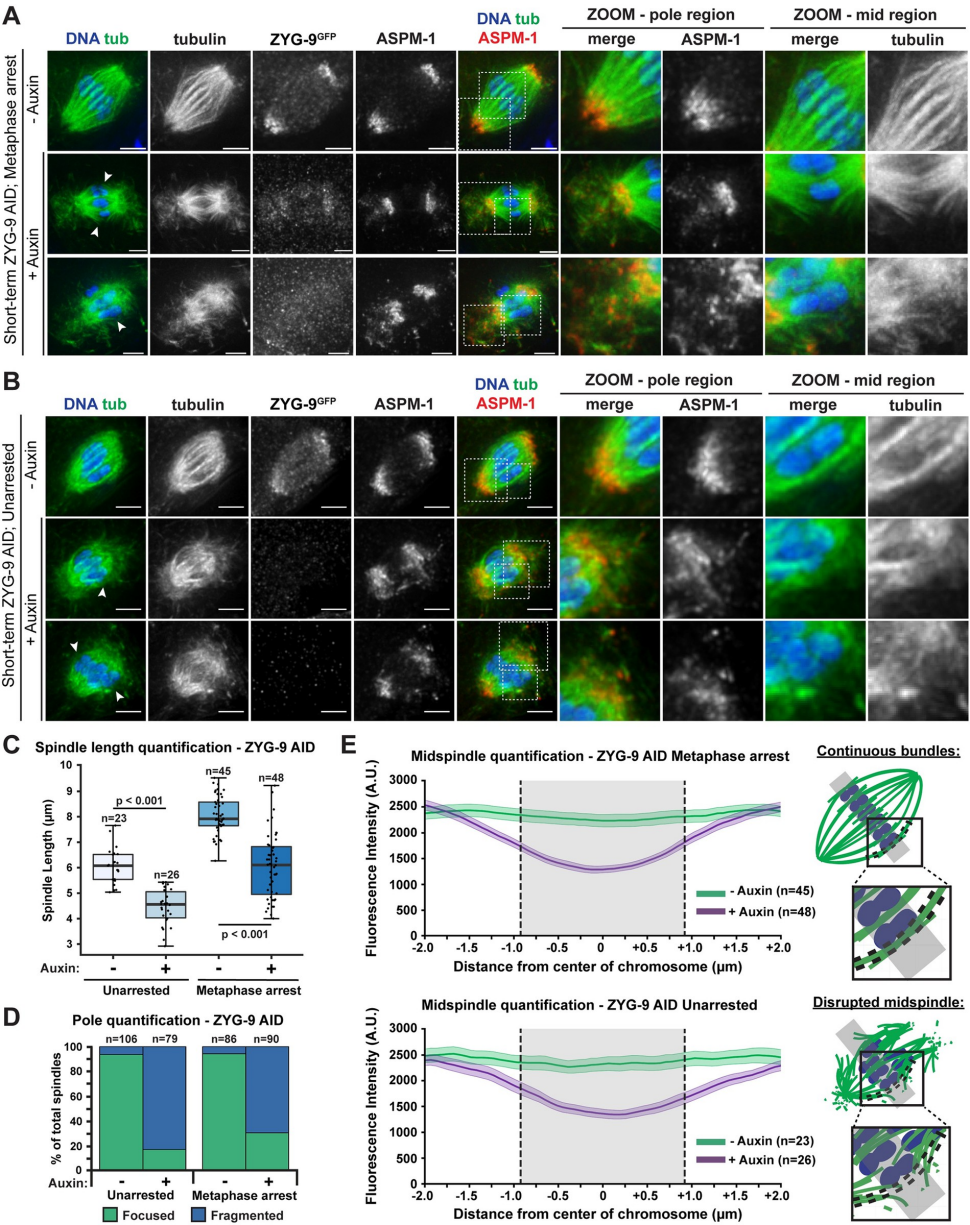
ZYG-9 depletion disrupts pole organization, midspindle bundle stability, and spindle length. (A, B) Spindles from Metaphase I-arrested *(emb-30(RNAi))* oocytes (A) or unarrested oocytes (B) treated with vehicle or 1mM auxin for 25–30 minutes. Shown are DNA (blue), tubulin (green), ASPM-1 (red), and ZYG-9 (stained with a GFP antibody; not shown in merge). In vehicle-treated oocyte spindles, the poles are tightly organized, and the microtubule bundles appear to cross from one side of the spindle to the other without interruption. In spindles from auxin-treated oocytes, the ZYG-9 signal is decreased and spindle pole integrity is compromised as shown by ASPM-1 and tubulin coming off the poles away from the spindle (zooms–pole region). Spindles from auxin-treated oocytes also showed defects in midspindle microtubules, where they appeared to terminate near the center of the spindle and splay away from the chromosomes (arrowheads, zooms–mid region). Bars = 2.5 μm. (C) Quantification of spindle length (pole to pole) in unarrested and Metaphase I-arrested oocytes treated with either vehicle or 1mM auxin. Box represents the first quartile, median, and third quartile. Whiskers extend to maxima and minima. Significance determined using a two-tailed t-test. n represents the number of spindles analyzed. (D, E) Quantification of pole phenotypes (D) or midspindle bundle splaying (E) from unarrested (vector control) and Metaphase I-arrested *(emb-30(RNAi))* oocytes treated with either vehicle or auxin. Schematic in (E) shows how midspindle defects were quantified. Shaded regions of lines represent +/- SEM, and gray shading represents average length of chromosome in that condition. n represents the number of spindles analyzed.

To examine the effects on pole morphology at higher resolution, we performed a short-term auxin treatment by soaking whole worms in auxin and then fixing Metaphase I-arrested oocytes for immunofluorescence. Protein depletion using this fixed imaging method is less rapid than in dissected oocytes [30]; we needed to incubate worms for 25–30 minutes in auxin prior to dissection to begin to see spindle phenotypes. This is likely because auxin needs additional time to reach the oocytes when these cells are not dissected, resulting in increased incubation times and milder phenotypes. This method confirmed that ZYG-9 is required to maintain the integrity of spindle poles; microtubules marked by ASPM-1 appeared to be dissociated from the pole following ZYG-9 depletion (Fig 3A and 3B, 63/90 spindles). In addition, we observed defects in microtubule organization in the middle region of the spindle (Fig 3A and 3B, arrowheads). In control oocytes, microtubule bundles run alongside chromosomes forming lateral associations [31]. Although these bundles are comprised of many short microtubules based on electron microscopy [4,32], at the resolution of light microscopy the bundles appear to run continuously across the center of the spindle (Fig 3A and 3B, top row). In contrast, following ZYG-9 depletion, microtubule bundles splayed away from the chromosomes (Fig 3A and 3B, rows 2–3); close examination of our lower resolution live imaging movies revealed similar disorganization of the mid-spindle region (Fig 2B, rows 2–6 white arrows, S6 and S7 Videos, n = 5). To quantify this splaying, we utilized curve-fitting linescans of bundles that ran laterally alongside chromosomes in the overlap zone (Fig 3E). In control spindles, midspindle bundles maintained a continuous line of fluorescence intensity in the vicinity of chromosomes. However, short-term ZYG-9 AID spindles had a significant decrease in microtubule fluorescence in close proximity to chromosomes, confirming the prevalence of microtubule bundle splaying in the midspindle region. Furthermore, we noted that ZYG-9-depleted spindles appeared significantly shorter than controls, and measured a substantial decrease of mean spindle length (~1.5 μm) in ZYG-9 depleted spindles (Fig 3C). Unarrested oocytes displayed the same spindle defects (Fig 3C–3E), demonstrating that they were not artifacts caused by the metaphase arrest. Finally, we observed some spindles that exhibited defects, even though ZYG-9 was still detectable by immunofluorescence (Fig 3A, second row), suggesting that ZYG-9 does not have to be fully depleted to disrupt spindle organization. Together, these data suggest that ZYG-9 is continuously required to maintain the integrity of the oocyte spindle and is especially important for the stability of acentrosomal poles.

### The TACC homolog TAC-1 is required for proper ZYG-9 localization to the meiotic spindle

**Fig 4:**
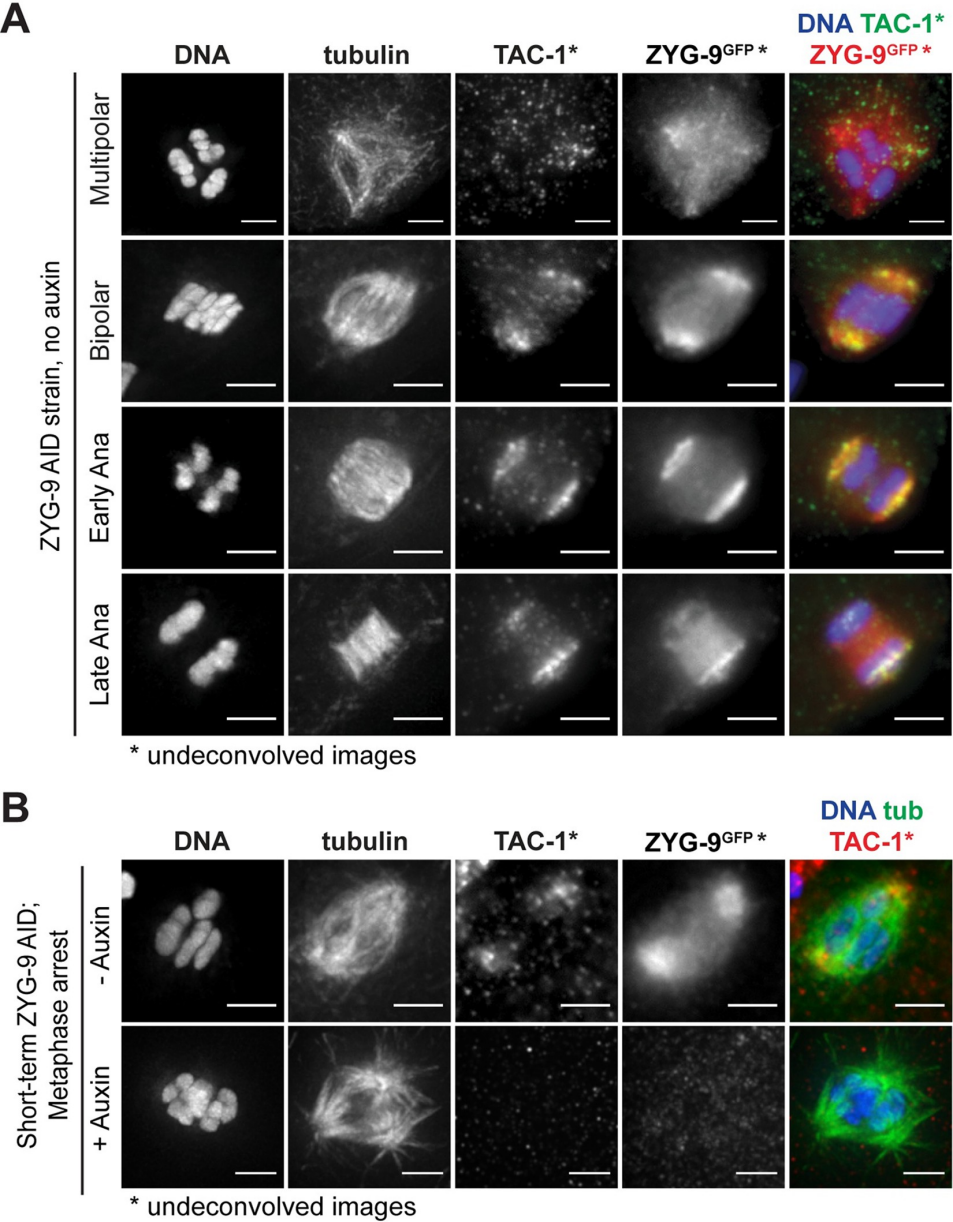
TAC-1 and ZYG-9 are interdependent for localization to acentrosomal poles. (A) IF imaging of oocyte spindles; shown are DNA (blue), TAC-1 (green), ZYG-9 (stained with a GFP antibody; red), and tubulin (not shown in merge). Colocalization of TAC-1 and ZYG-9 is evident in metaphase and persists throughout anaphase (12/12 spindles). DNA and tubulin channels were deconvolved, while ZYG-9 and TAC-1 channels were not due to higher background staining of the TAC-1 antibody. Bars = 2.5μm. (B) IF imaging of oocyte spindles in the ZYG-9 AID strain in Metaphase I-arrest *(emb-30(RNAi))* conditions; shown are DNA (blue), tubulin (green), TAC-1 (red), and ZYG-9 (stained with a GFP antibody; not shown in merge). Short-term ZYG-9 depletion via addition of auxin disrupts localization of TAC-1 to acentrosomal poles (8/8 spindles). As in (A), ZYG-9 and TAC-1 channels were not deconvolved. Bars = 2.5μm.

Next, we sought to investigate how ZYG-9 is regulated in oocyte meiosis. During mitosis, ZYG-9 forms a complex with the transforming acidic coiled-coil (TACC) homolog TAC-1 and these proteins are interdependent for localization to centrosomes [14,33]. Moreover, TAC-1 and ZYG-9 both localize at acentrosomal poles [6]. To study the relationship between these two proteins in more detail, we generated an antibody against the C-terminus of TAC-1, validated it using immunofluorescence (S2 Fig), and then used it to visualize TAC-1 and ZYG-9 localization simultaneously in oocytes. TAC-1 localization was difficult to discern on forming multipolar spindles using our antibody, but once bipolarity was established, TAC-1 clearly colocalized with ZYG-9 at acentrosomal poles and this colocalization persisted throughout anaphase (Fig 4A, 14/15 oocytes). Notably, when ZYG-9 was acutely depleted from Metaphase I arrested spindles, TAC-1 localization to poles was also disrupted (Fig 4B, 8/8 spindles), suggesting that ZYG-9 is required to maintain TAC-1 at spindle poles.

**Fig 5:**
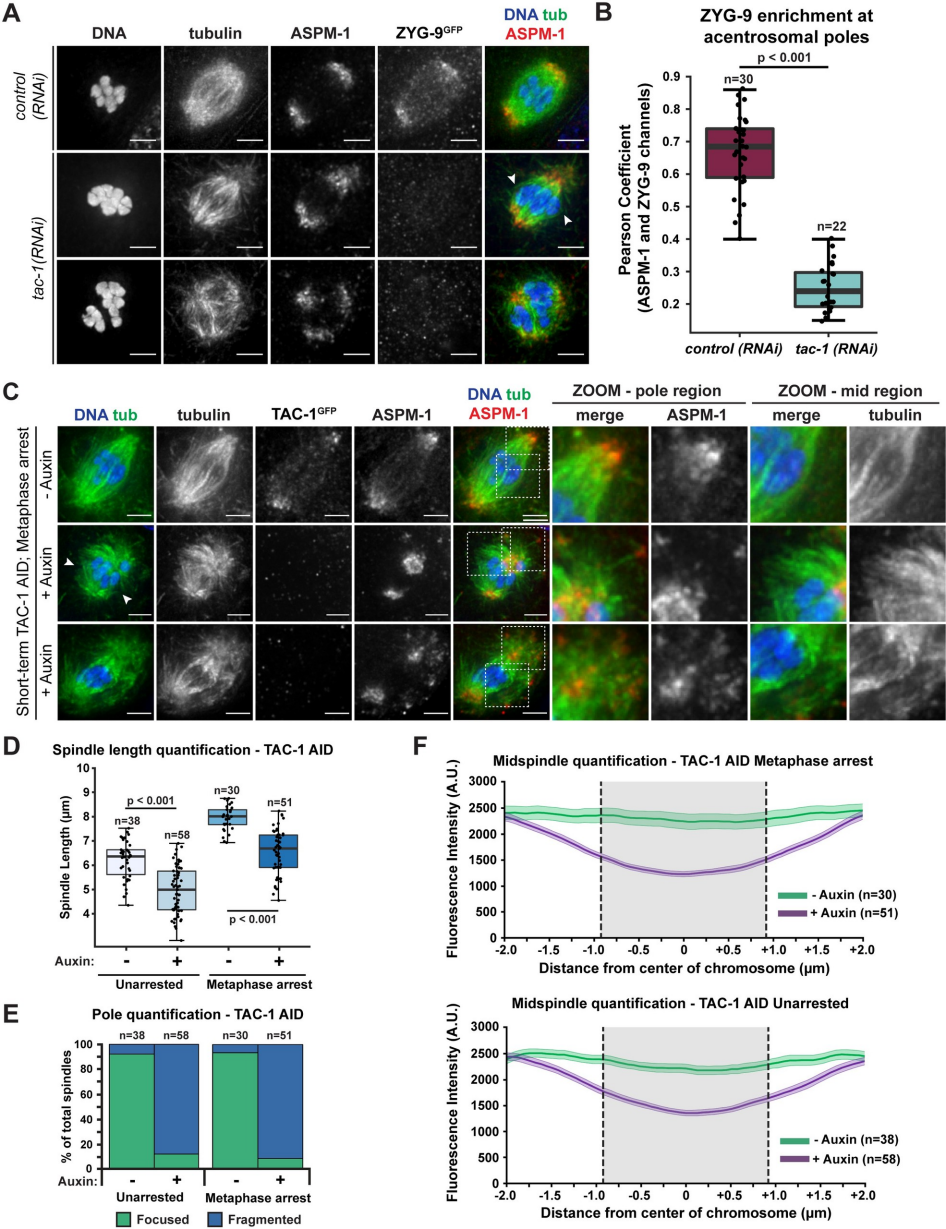
TAC-1 is required for proper ZYG-9 localization to oocyte meiotic spindles. (A) IF imaging of oocyte spindles in the ZYG-9 AID strain in either control or *tac-1(RNAi)* conditions. Shown are DNA (blue), tubulin (green), ASPM-1 (red), and ZYG-9 (stained with a GFP antibody; not shown in merge). ZYG-9 enrichment at acentrosomal poles is lost in most spindles observed (20/22 spindles), and some spindles appear to have lost all ZYG-9 localization to the meiotic spindle; midspindle disruption is highlighted with arrowheads. Bars = 2.5μm. (B) Quantification of ZYG-9 enrichment at poles from oocyte spindles observed in (A); a Pearson coefficient was calculated for each image by comparing the ZYG-9 and ASPM-1 channels. Total spindles measured in each condition are noted above box plots; boxes represent first quartile, median, and third quartile. (C) IF imaging of metaphase-arrested oocyte spindles in the TAC-1 AID strain. Shown are DNA (blue), tubulin (green), ASPM-1 (red), and TAC-1 (stained with a GFP antibody; not shown in merge). In control oocytes, the poles are tightly organized and the midspindle microtubule bundles appear stable across the overlap zone. In auxin-treated oocytes, the TAC-1 signal is lost and spindle phenotypes are identical to those observed in ZYG-9 AID. Spindle pole integrity is compromised as shown by the ASPM-1 and tubulin coming off the poles (zooms–pole region) and midspindle microtubule bundles terminate near the center of the spindle and splay away from the chromosomes (arrowheads, zooms–mid region). Bars = 2.5 μm. (D) Quantification of spindle length (pole to pole) in unarrested and Metaphase I-arrested oocytes treated with either vehicle or 1mM auxin. Box represents the first quartile, median, and third quartile. Whiskers extend to maxima and minima. Significance determined using a two-tailed t-test. n represents the number of spindles analyzed. (E, F) Quantification of pole phenotypes (E) or midspindle splaying (F) from unarrested (vector control) and Metaphase I-arrested *(emb-30(RNAi))* oocytes treated with either vehicle or 1mM auxin. Shaded regions of lines represent +/- SEM, and gray shading represents average length of chromosome in that condition. n represents the number of spindles analyzed.

To determine if TAC-1 also promotes the enrichment of ZYG-9 at acentrosomal poles, we imaged ZYG-9 localization in oocytes following *tac-1(RNAi)* (Fig 5A). ZYG-9 enrichment was lost at the poles in a majority of oocyte spindles (20/22) (Fig 5A, rows 2 and 3, Fig 5B). Moreover, the spindle defects following *tac-1(RNAi)* were nearly identical to ZYG-9 depletion phenotypes, including fragmented poles with dispersed ASPM-1, disruption of midspindle microtubules (Fig 5A, arrowheads), and the formation of multipolar spindles (Fig 5A, row 3).

Because we found that TAC-1 is required for robust ZYG-9 localization to the meiotic spindle and acentrosomal poles, we hypothesized that the *tac-1(RNAi)* phenotypes could be primarily explained by disruption of ZYG-9. To test this, we performed short-term ZYG-9 AID depletion in worms treated with *tac-1(RNAi)*, reasoning that if the spindle phenotypes worsened then it would suggest that TAC-1 has functions beyond recruiting ZYG-9 to the spindle. However, after treating *tac-1(RNAi)* ZYG-9 AID worms with auxin, oocyte spindles appeared morphologically similar to either *tac-1(RNAi)* or ZYG-9 AID alone (S3A Fig). ASPM-1 labeling indicated clear pole fragmentation (77/94 with auxin alone, 45/51 with *tac-1* RNAi and auxin) and a majority of spindles contained splayed midspindle microtubule bundles (S3A Fig, arrowheads, 73/94 with auxin alone, 37/51 with *tac-1(RNAi)* and auxin), and the percentages of pole fragmentation and midspindle disruption between these three depletion conditions were relatively similar (S3B and S3C Fig). Together, these data support the hypothesis that a major role of TAC-1 in oocyte meiosis is to regulate ZYG-9 localization or stability.

To further explore the connection between TAC-1 and ZYG-9, we sought to acutely remove TAC-1 from pre-formed spindles and compare the phenotype to acute ZYG-9 AID. To this end, we generated a TAC-1 AID strain by inserting a GFP::degron tag at the endogenous *tac-1* locus. To validate this strain, we first performed long-term AID-mediated depletion to confirm that this recapitulated *tac-1(RNAi)* phenotypes. One-cell stage embryos from TAC-1 AID worms grown on auxin plates had clear mitotic spindle positioning defects with shorter astral microtubules (S4A Fig, 14/14 embryos), and oocyte spindles exhibited pole fragmentation and splaying of midspindle microtubule bundles (S4B Fig, 51/53 spindles), similar to *tac-1(RNAi)* [14,33] (Fig 5A). We also observed a clear loss of TAC-1 signal using immunofluorescence (S4A and S4B Fig, 100% of spindles), and confirmed efficient depletion using western blotting (S4C Fig). Finally, we did not observe substantial embryonic lethality in the absence of auxin (0.43% +/- 0.4% lethality), suggesting that introduction of the GFP::degron tag was not affecting TAC-1 function, while growth on auxin-containing plates increased lethality to 98.6% (+/- 0.7%), consistent with previous reports of *tac-1(RNAi)* [13].

After validating our TAC-1 AID strain, we used short-term AID to deplete TAC-1 from stable bipolar spindles. The majority of spindles exhibited pole fragmentation and midspindle defects indistinguishable from short-term AID depletions of ZYG-9; small microtubule fragments with ASPM-1 foci could be seen dissociating from acentrosomal poles (Fig 5C, columns 6–7) and splaying of microtubule bundles occurred in the vicinity of chromosomes (Fig 5C, columns 8–9). Quantifications revealed similar penetrance of phenotypes compared to ZYG-9 depletions, and mean spindle length also significantly decreased (~1.5μm) in both unarrested and metaphase-arrested conditions (Fig 5D–5F). Together, these results suggest that TAC-1 is required for maintaining spindle integrity, and further support our hypothesis that TAC-1’s primary role in oocyte meiosis is to promote proper ZYG-9 activity.

### ZYG-9 and TAC-1 are not required to form or stabilize spindle poles in all contexts

Next, we sought to investigate how ZYG-9 promotes acentrosomal spindle stability. Since ZYG-9 localizes to spindle poles (Figs 1B, 1C, S1A and S1B) and is required for both pole coalescence [6] (Fig 1D) and maintenance (Figs 2 and 3), one possibility is that a population of ZYG-9 at spindle poles functions to organize microtubule minus ends into a stable structure. However, since ZYG-9 depletion also causes microtubule bundles in the center of the spindle to splay (Figs 2 and 3) and decreases spindle length (Figs 3C and 5D), it is possible that the pole defects could instead be the result of a more global effect on spindle microtubule stability or dynamics. For instance, if ZYG-9 depletion affects microtubules in the center of the spindle, disruption of this midspindle region could destabilize the entire structure by disrupting the microtubule crosslinking and sorting forces provided by microtubule motors and MAPs. Consistent with this second possibility, we observed a substantial population of ZYG-9 in the center of the spindle (Fig 1B). Notably, not all spindle pole components have this midspindle population, as the intensity of ASPM-1 fluorescence is much less prominent in this region (Fig 1C).

**Fig 6:**
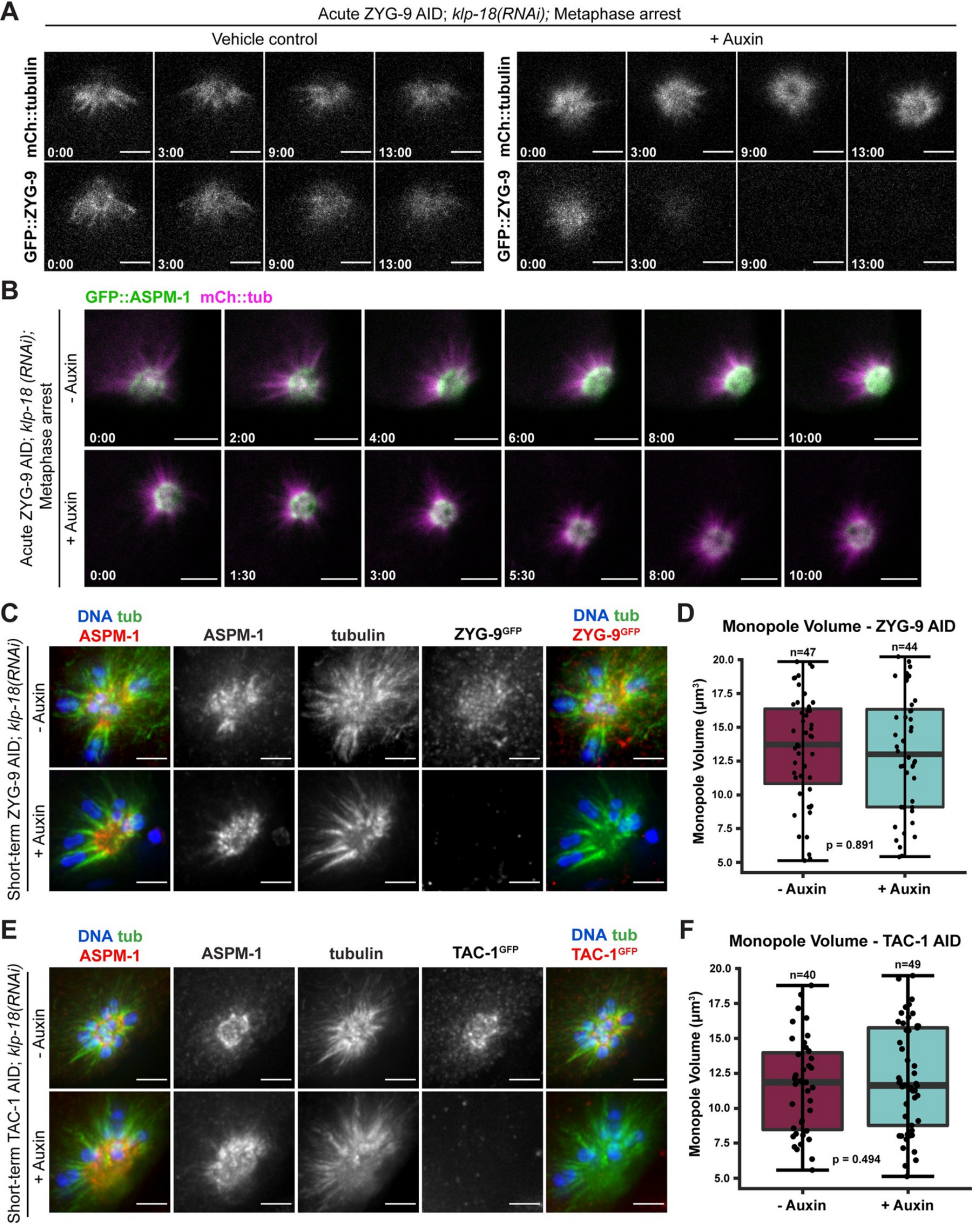
ZYG-9 and TAC-1 are not required for acentrosomal pole stability of monopolar spindles. (A) Movie stills from Metaphase I-arrested *klp-18(RNAi))* oocytes expressing mCherry::tubulin and degron::GFP::ZYG-9 acutely treated with either vehicle (left, n = 3) or 100μM auxin (right, n = 3). Auxin treatment causes a rapid loss of ZYG-9 signal but the structure of the monopolar spindle does not noticeably change. Bars = 5 μm. Timestamp = min:sec. (B) Movie stills from Metaphase I-arrested *klp-18(RNAi))* oocytes expressing mCherry::tubulin, degron::GFP::ZYG-9, and GFP::ASPM-1. Whether oocytes were dissected into vehicle (left, n = 5) or 100μM auxin (right n = 5) solutions, the integrity of the monopole (as seen by ASPM-1) was not compromised; pole morphology and spindle structure remains stable throughout the timelapses. Bars = 5 μm. Timestamp = min:sec. (C-F) Examination of pole morphology following ZYG-9 or TAC-1 depletion. (C, E) IF imaging of oocyte spindles in the ZYG-9 AID strain (C) or the TAC-1 AID strain (E) in *klp-18(RNAi)* conditions; shown are DNA (blue), microtubules (green), ZYG-9 or TAC-1 (stained with a GFP antibody; red, merge on the right), and ASPM-1 (red, merge on the left). When depleting either ZYG-9 or TAC-1 via short-term auxin treatment, monopolar spindles appear unperturbed, as seen through a singular, focused pole of ASPM-1. Bars = 2.5μm. (D, F) Quantification of IF imaging represented in (C and E); there is no significant difference in the volume of monopoles between untreated and auxin-treated conditions when either ZYG-9 (D) or TAC-1 (F) is depleted. Box represents the first quartile, median, and third quartile. Whiskers extend to maxima and minima. Significance determined using a two-tailed t-test. n represents the number of spindles analyzed.

Consequently, to explore these possibilities we asked whether ZYG-9 depletion would affect the formation of monopolar spindles. These structures have a single pole and therefore lack the plus end overlap region that is present at the center of bipolar spindles. Thus, if monopoles are disrupted following ZYG-9 depletion, it would suggest that ZYG-9 has a structural role at poles, rather than the pole defects in bipolar spindles arising through disruption of the central overlap zone. To generate monopolar spindles, we depleted KLP-18^kinesin-12^ via RNAi in the ZYG-9 AID strain; in oocytes lacking KLP-18, microtubule minus ends fail to be sorted outwards during spindle assembly and instead organize into a single ASPM-1-marked pole [3,31]. We then acutely depleted ZYG-9 by dissecting metaphase-arrested ZYG-9 AID, *klp-18(RNAi)* oocytes expressing mCherry::tubulin and degron::GFP::ZYG-9 into auxin. As ZYG-9 signal decreased, monopolar spindles remained stable and maintained their aster-like structure, similar to control spindles where ZYG-9 was present (Fig 6A and S8 and S9 Videos, n = 3). As a complementary approach, we repeated this experiment in a strain also expressing GFP::ASPM-1 to assess pole morphology. Upon dissection into auxin, we noted a slight decrease in GFP fluorescence (due to ZYG-9 depletion), but this depletion did not cause any noticeable effects on the organization of microtubules or ASPM-1 (Fig 6B and S10 and S11 Videos, n = 5). To support these observations, we depleted ZYG-9 in the absence of metaphase arrest, and performed immunofluorescence to assess spindle and pole morphology at higher resolution; we then measured the volume of ASPM-1 labeling as a proxy for pole organization. There was no apparent change in spindle morphology or ASPM-1 volume when ZYG-9 was depleted from monopolar spindles (p = 0.891) (Fig 6C and 6D). Notably, we obtained nearly identical results when we performed analogous experiments on TAC-1. Acute TAC-1 depletion did not cause any noticeable changes in monopole morphology (Fig 6E and 6F). Moreover, the mean ASPM-1 volumes did not significantly change upon auxin treatment (p = 0.494), and were consistent with volumes measured following ZYG-9 depletion. Thus, ZYG-9 is not required to maintain acentrosomal pole structure *per se*, and instead performs a function that is specifically required for the formation and stabilization of bipolar spindles. These results, in combination with the midspindle defects observed following ZYG-9 depletion from bipolar spindles (Fig 3A and 3E), suggest that ZYG-9 does not solely function at acentrosomal spindle poles, but rather promotes pole stability through more global effects on spindle dynamics or organization.

### ZYG-9 is highly dynamic at acentrosomal spindle poles

While centrosomes act as structural cues that organize mitotic spindle poles, it is not clear whether there are factors that stably associate with acentrosomal poles and perform this scaffolding role in *C*. *elegans* oocytes. Given that our data suggest that ZYG-9 stabilizes poles through a global effect on spindle microtubules, we would not expect it to act as this type of static scaffold. However, since nothing is known about the dynamics of acentrosomal pole proteins in *C*. *elegans*, we set out to test this prediction directly.

**Fig 7:**
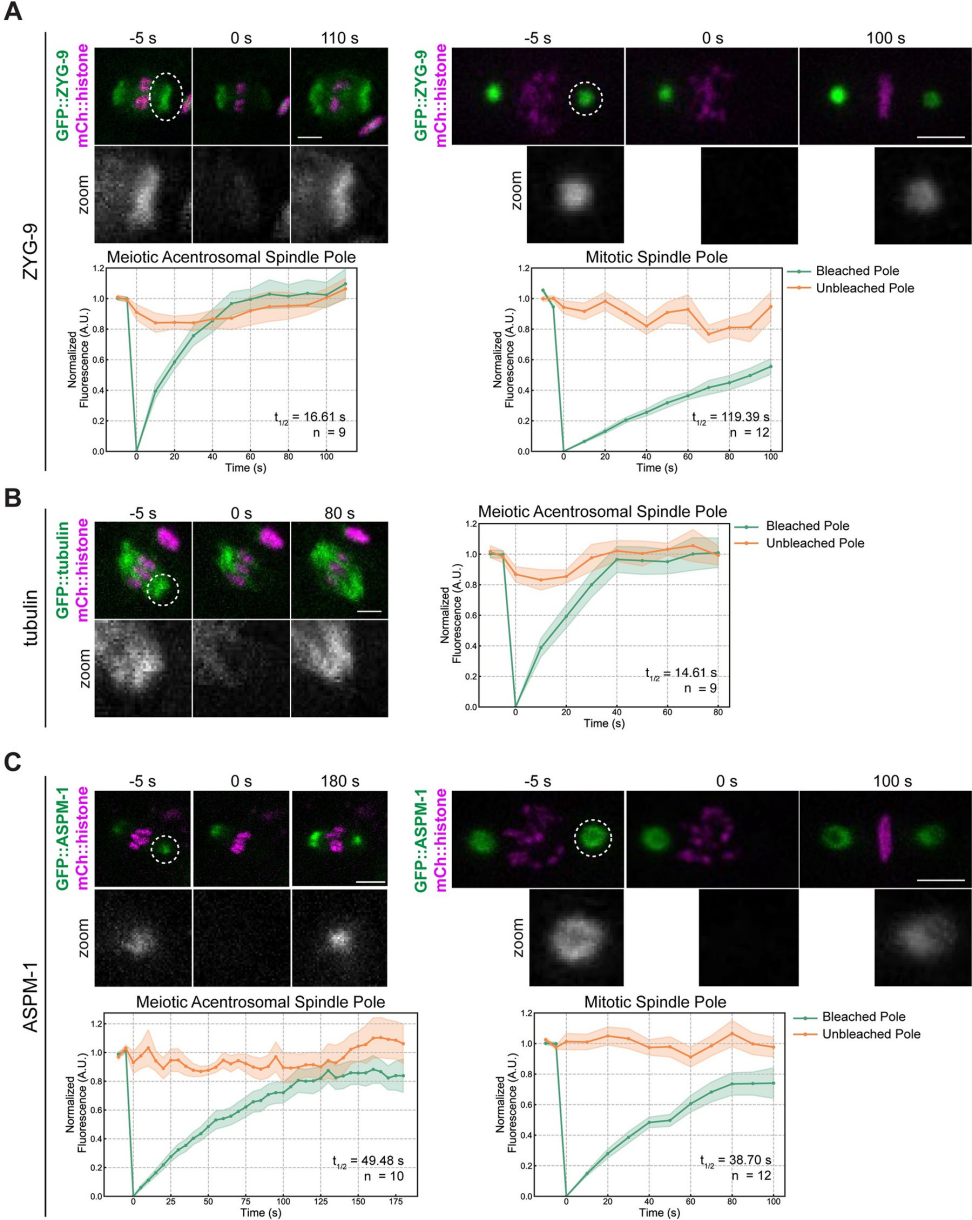
FRAP analysis of ZYG-9 and ASPM-1 at acentrosomal poles compared to at centrosomes. (A-C) FRAP recovery curves and stills from movies for ZYG-9 (A), tubulin (B), and ASPM-1 (C) at meiotic and mitotic spindle poles (except for tubulin; only recovery at the meiotic spindle is shown). In graphs, the bleached pole curve is green, the unbleached pole curve is orange, the solid lines are the average, and the standard error of the mean is shaded. For all images, the specified bleached protein is in green and the chromosomes are in magenta. Zooms show only the bleached region. t_1/2_ was calculated from fitting the recovery curves to a single exponential function (Materials and Methods); see S5 Fig. n represents the number of spindles analyzed to generate each curve; movies for each condition were obtained across several days so represent multiple biological replicates. ZYG-9 is highly dynamic at acentrosomal spindle poles, displaying a similar recovery time to tubulin, while ASPM-1 turns over less rapidly and has similar dynamics at centrosome-containing and acentrosomal poles. Bars = 2.5μm (meiosis) and 5μm (mitosis).

A previous study assessed ZYG-9 dynamics at the poles of mitotic centrosome-containing spindles using fluorescence recovery after photobleaching (FRAP) [34]. That investigation demonstrated that the exchange of ZYG-9 between the centrosome and cytoplasm was not highly dynamic, suggesting that it is a relatively stable component of centrosome-containing poles. To determine if this is also true at acentrosomal spindle poles, we performed an analogous experiment by photobleaching GFP::ZYG-9 and assessing its recovery at the poles of oocyte spindles compared to at centrosomes (Figs 7A and S5A). To assess the stability of ZYG-9 in mitosis we imaged centrosomes in the EMS (endomesodermal precursor) cell of 4-cell embryos since these cells remain stable at metaphase for a few minutes, allowing us to image fluorescence recovery [34,35]. This experiment revealed that ZYG-9 turns over much more rapidly at acentrosomal spindle poles (t_1/2_ = 16.61s) compared to centrosomes (t_1/2_ = 119.39s) during metaphase (Figs 7A and S5A and S12 and S13 Videos). Furthermore, ZYG-9 fluorescence at acentrosomal poles recovered to pre-bleach levels during the time course whereas ZYG-9 fluorescence at centrosomes did not. Strikingly, the rate of ZYG-9 turnover at acentrosomal poles was so rapid that it was similar to the recovery of tubulin (t_1/2_ = 14.61s) (Figs 7B and S5B and S14 Video). These results suggest that ZYG-9 is not a stable component of acentrosomal poles and moves dynamically throughout the spindle.

Following on these findings, we sought to investigate whether the faster ZYG-9 turnover in oocytes was the result of the inherent organization of acentrosomal poles, rather than reflecting a specific difference in ZYG-9 behavior; if this were the case, other pole components would also show the same discrepancy in dynamics compared to centrosomes. To test this, we performed FRAP analysis on ASPM-1, another pole-enriched microtubule binding protein, and found that this protein had relatively similar turnover rates both in oocytes and in mitotically-dividing embryos (t_1/2_ = 49.48s and 38.70s, respectively) and the fluorescence recovered to similar levels during the time of filming (Figs 7C and S5C and S15 and S16 Videos). Moreover, ASPM-1 recovered more slowly than ZYG-9 and tubulin (Figs 7 and S5), demonstrating that not all pole proteins display the rapid turnover seen for ZYG-9 at acentrosomal poles, and suggesting that ASPM-1 may be part of more stabilized pole structure. Therefore, the rapid turnover of ZYG-9 in oocytes is not solely due to differences in pole organization in the absence of centrosomes. Rather, the faster ZYG-9 dynamics imply that ZYG-9 does not play a static, structural role at acentrosomal poles, and instead support the idea that ZYG-9 acts more globally to regulate spindle stability during oocyte meiosis.

## Discussion

### ZYG-9 is required to stabilize and maintain acentrosomal poles during oocyte meiosis

**Fig 8:**
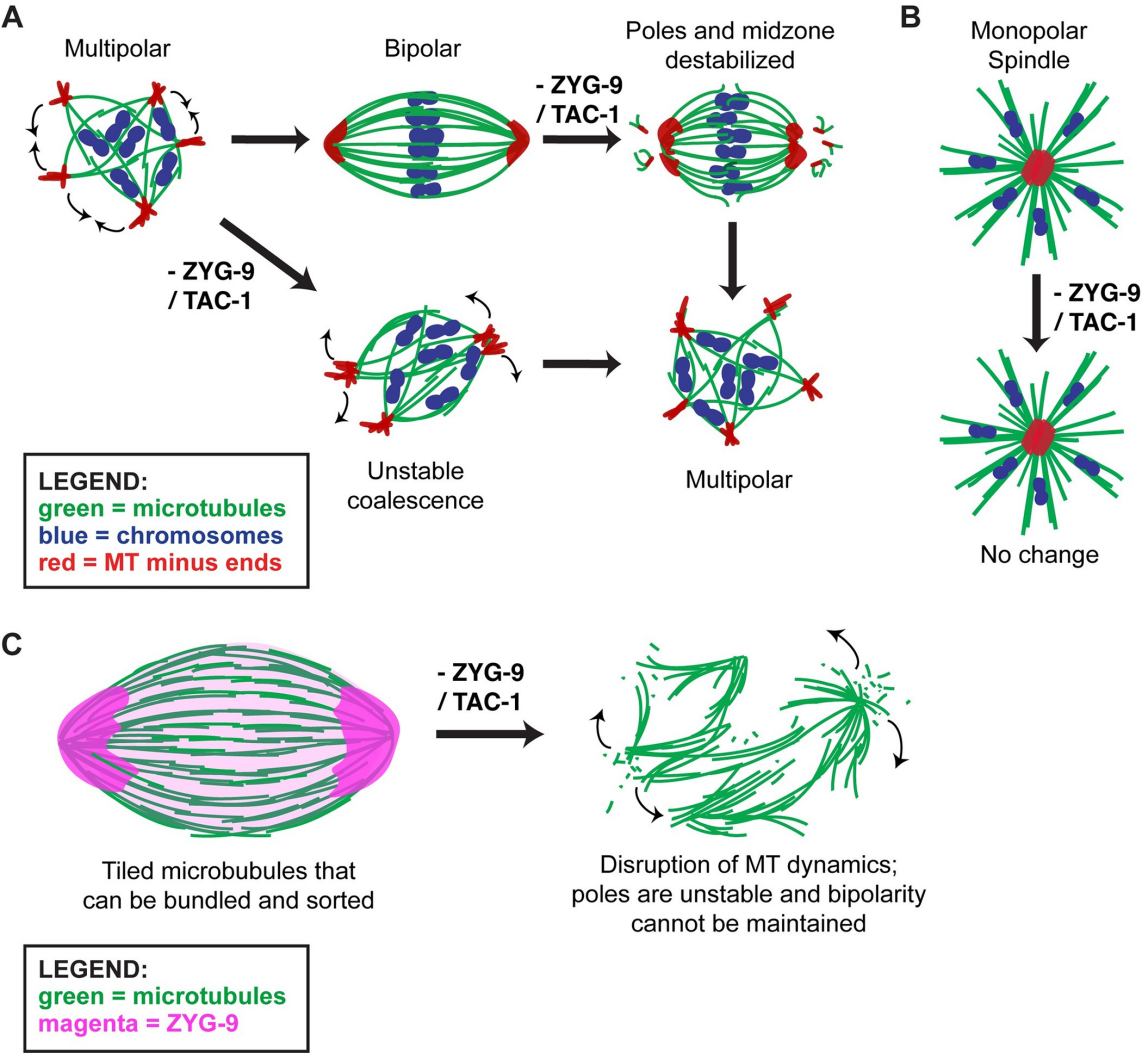
Model of acentrosomal pole coalescence and stability provided by ZYG-9. (A) ZYG-9 is required to establish and maintain acentrosomal pole stability during meiosis. Removal of ZYG-9 prior to spindle assembly prevents multipolar spindles from stably coalescing into bipolar spindles. Additionally, removing ZYG-9 from stable bipolar spindles causes splaying of microtubule bundles near chromosomes and fragmentation of acentrosomal poles, ultimately causing the spindle to lose bipolarity and revert to a multipolar state. (B) Removal of ZYG-9 from a monopolar spindle causes no obvious defects to spindle morphology or monopole stability, suggesting that ZYG-9’s role in spindle bipolarity is not tied directly to acentrosomal poles. (C) Model: ZYG-9 is required for proper microtubule dynamics within the acentrosomal spindle, which is critical for the organization of tiled microtubules into stable bundles via microtubule motors/MAPs. Removal of ZYG-9 leads to defects in microtubule stabilization, leading to rapid splitting of acentrosomal poles, splaying of midspindle microtubule bundles, and loss of spindle bipolarity.

Taken together, our data support a model in which ZYG-9 provides spindle stability throughout meiosis (Fig 8A). ZYG-9 was previously shown to localize to the spindle as multiple poles are formed; these poles then merge, forming a stable bipolar structure. Without ZYG-9 present, nascent poles marked by ASPM-1 form, but can never stably coalesce [6]. Our current study now extends these findings, demonstrating that ZYG-9 is not only essential for spindle assembly but is also continuously required to maintain spindle stability. We found that when ZYG-9 was removed from a pre-formed spindle, acentrosomal poles destabilize and the overlap zone of the spindle is disrupted, causing the spindle to become unstable and ultimately lose bipolarity. Similar results were recently reported in a study using temperature-sensitive mutants, confirming an essential role for ZYG-9 in stabilizing the acentrosomal spindle [36].

Importantly, the observation that ZYG-9 depletion does not affect monopolar spindle structure demonstrates that the pole fragmentation phenotype does not occur in all contexts (Fig 8B). Thus, ZYG-9 is not required for pole formation *per se*, and instead likely regulates spindle stability more globally. This interpretation is supported by work on ZYG-9 homologs in other organisms. The *Xenopus* homolog of ZYG-9, XMAP215, has been demonstrated to have microtubule polymerase activity; this protein utilizes multiple TOG domains to bind tubulin dimers for incorporation into growing microtubule plus ends [20,24,37,38]. *C*. *elegans* ZYG-9 contains three of these TOG domains, and thus is also likely to have polymerase activity. Consistent with this hypothesis, a recent study demonstrated that a minimal condensate comprised of ZYG-9, the microtubule associated protein TPXL-1, and the centrosomal protein SPD-5 can generate microtubule asters *in vitro*; this activity required functional TOG domains [34]. Moreover, a role in promoting polymerization is also consistent with the *in vivo* phenotypes of ZYG-9 depletion; astral microtubules are substantially shorter following ZYG-9 depletion in mitotically-dividing embryos [14,26,33], and we demonstrated in this study that oocyte spindles shorten following acute ZYG-9 removal, before they lose bipolarity.

Together, these data suggest a model that ZYG-9 acts to maintain a stable population of microtubules within the acentrosomal spindle in *C*. *elegans* oocytes (Fig 8C). Previous EM imaging revealed that the oocyte meiotic spindle is comprised of a dense population of short, tiled microtubules rather than continuous long filaments [4,32]. It is therefore likely that the oocyte must carefully regulate microtubule polymerization rates, in order to maintain the appropriate microtubule substrate for motors that drive spindle formation. We previously found that the kinesins KLP-18 and BMK-1 sort microtubule minus ends outwards, where their ends can then be coalesced into stable poles by cytoplasmic dynein [3,8]. We therefore propose that ZYG-9 promotes the stability of microtubules that these motors and other MAPs can act upon; without ZYG-9 activity, overlapping antiparallel microtubule bundles would not be properly stabilized and outwards sorting force would no longer be bidirectional, resulting in splitting of acentrosomal poles into a disorganized multipolar spindle (Fig 8C). It has been demonstrated that a stable bipolar spindle in a *mei-1* temperature sensitive mutant oocyte loses spindle organization within minutes of shifting to the restrictive temperature and losing microtubule severing activity [11], highlighting that tight regulation of spindle dynamics is critical for maintaining bipolarity.

However, it is also plausible that ZYG-9 and TAC-1 negatively regulate microtubule growth and ectopic pole formation, as proposed in two recent studies. First, depletion of ZYG-9 via RNAi led to a notable increase in ectopic microtubule asters throughout oocytes [6]. In addition, rapid upshifts and downshifts to/from restrictive temperatures with *zyg-9* temperature sensitive mutants revealed that ASPM-1 foci grew larger in volume and intensity when ZYG-9 was inactivated, and reverted to control levels upon downshifting [36]; these results raise the possibility that ZYG-9 activity suppresses microtubule growth and pole formation instead of promoting it.

Regardless of whether ZYG-9 promotes or restricts microtubule growth in acentrosomal spindles, it stands to reason that ZYG-9 functions by regulating some aspect of microtubule dynamics. In addition to its characterized polymerase activity, recent work demonstrated that XMAP215 can also promote microtubule catastrophe despite rapid polymerization of plus ends [22]. Moreover, XMAP215 has been shown to promote microtubule nucleation through an interaction with the ɣ-tubulin ring complex [21]. Further investigation will be invaluable for evaluating how ZYG-9 affects microtubule dynamics in *C*. *elegans* oocytes and for better understanding how the concerted activities of MAPs and motors generate stable bipolarity in the absence of centrosomes.

### TAC-1 is essential for proper ZYG-9 function

In addition to gaining insight into the role of ZYG-9, we also demonstrated that ZYG-9 and TAC-1 are interdependent for proper localization to the meiotic spindle, similar to what has been observed in *C*. *elegans* mitotically-dividing embryos [14,33]. Depletion of TAC-1 using RNAi led to loss of ZYG-9 localization to acentrosomal poles, and acute removal of TAC-1 from stable bipolar spindles phenocopied rapid ZYG-9 depletion. Furthermore, concurrent depletion of ZYG-9 and TAC-1 did not exacerbate the spindle phenotypes when compared to ZYG-9 AID depletions alone. These data suggest that the primary function of TAC-1 is to promote proper ZYG-9 localization. TAC-1 could perform this function by directly targeting ZYG-9 to the meiotic spindle, by stabilizing ZYG-9 (as has been shown in mitosis [14]), or by both mechanisms. We speculate that this interaction could spatially regulate microtubule polymerase activity, promoting microtubule growth locally but minimizing potential ectopic growth throughout the cytoplasm. When disrupting ZYG-9/TAC-1 function via fast-acting *ts* mutants, a recent study reported an overall increase in microtubules within Meiosis I oocytes in *tac-1* mutants, but not *zyg-9* mutants [36]. This observation is consistent with the hypothesis that TAC-1 interacts with ZYG-9 to constrict where microtubule polymerization can occur during the meiotic divisions.

In the future it would be interesting to further investigate how the interaction between ZYG-9 and TAC-1 is regulated. In *Xenopus* egg extracts, the TAC-1 homolog Maskin is phosphorylated by Eg2 (Aurora A kinase) and this phosphorylation regulates Maskin’s localization and function [39]. Related studies of mitotic *Drosophila* embryos have demonstrated that Aurora A stabilizes the interaction between TAC-1 and ZYG-9 homologs (D-TACC and Msps, respectively) [40]. The *C*. *elegans* Aurora A ortholog, AIR-1, is essential for spindle assembly and microtubule nucleation in oocytes [41], raising the possibility that it could work in concert with ZYG-9. However, whether Aurora A regulates the interaction between TAC-1 and ZYG-9 in *C*. *elegans* oocytes remains to be investigated. During mitosis, ZYG-9 and TAC-1 have also been shown to form a complex with another kinase, ZYG-8 [13]. ZYG-8 mutants were shown to have similar mitotic defects as those seen with ZYG-9 and TAC-1 depletion [13,42], but the function of ZYG-8 during oocyte meiosis has not been extensively characterized. A previous study observed defects in anaphase spindle elongation with removal of ZYG-8 activity in a temperature-sensitive mutant, suggesting a decrease in microtubule polymerization [43], but no spindle assembly or stability phenotypes were noted prior to anaphase. It is possible that ZYG-8 is responsible for phosphorylating TAC-1 in *C*. *elegans* oocyte meiosis to stabilize the interaction between TAC-1 and ZYG-9, rather than Aurora A. If phosphorylation of TAC-1 by ZYG-8 stabilizes the interaction of TAC-1 and ZYG-9 and their localization to the meiotic spindle, loss of ZYG-8 activity could lead to loss of ZYG-9 polymerase activity, which could account for the reduced anaphase spindle elongation previously observed. Future experiments will be important to determine if ZYG-8 is interacting with ZYG-9/TAC-1, to clarify its role in oocyte meiosis.

### Acentrosomal pole proteins have distinct and separate contributions to pole coalescence and stability

Our work supports a crucial role for ZYG-9 in maintaining the integrity of acentrosomal poles. However, in the future it will be important to define the exact hierarchal structure of proteins that act to assemble the pole and then to coalesce and stabilize these structures. While localization of numerous proteins to acentrosomal poles has been reported (reviewed in [44,45]), how each protein contributes to pole organization, coalescence, and maintenance remains a crucial gap in knowledge.

Through FRAP of acentrosomal spindle pole components, we have begun to tease apart differences in the dynamics of pole proteins, laying the foundation for a more comprehensive analysis that could shed light on the nature of acentrosomal poles. Notably, ZYG-9 recovered from bleaching in the same timeframe as tubulin itself, suggesting that ZYG-9 is extremely dynamic on the acentrosomal spindle. This is in stark contrast to another pole protein, ASPM-1, which took much longer to recover. The slow ASPM-1 recovery was surprising, and may provide the first evidence that the acentrosomal pole in *C*. *elegans* oocytes is not simply a collection of dynamic microtubules stitched together by motor proteins; it is possible that some components form a more lasting pole structure. This finding opens the door for analysis of other known acentrosomal pole proteins, to distinguish between factors that may also have a more stable pole association versus those that transiently localize there to play a broader role in spindle organization or dynamics. In mammalian oocytes, some pole proteins have been shown to form a liquid-like spindle domain that has been proposed to concentrate microtubule regulatory factors to promote spindle formation [46]. In *C*. *elegans*, centrosomes have been shown to be selective condensates that nucleate microtubule arrays by concentrating tubulin subunits [34], but the nature of acentrosomal poles in this system is not understood. Our experiments comparing the dynamics of proteins at centrosomal and acentrosomal poles in the same organism highlight a new way for dissecting the differential roles of known MAPs and motors in different contexts, to shed light on the differences between poles with and without centrosomes.

Another powerful approach going forward will be to use the degron system to acutely remove proteins from pre-formed bipolar and monopolar oocyte spindles, as we did in this study for ZYG-9, to compare the depletion phenotypes across different pole proteins. In a recent study, we used these same assays to investigate dynein and found that, like ZYG-9, dynein is required for both pole organization and stability [8]. However, the phenotype of dynein depletion was markedly different from what we report for ZYG-9 depletion in the current study; while acute dynein depletion caused focused poles to splay, the center of the spindle remained intact and therefore the spindles were able to maintain bipolarity. Moreover, when we depleted dynein from monopolar spindles, the entire monopole came apart, dispersing microtubule bundles into the cytoplasm. These findings suggest that in contrast to ZYG-9, dynein plays a specific role at the pole itself, acting to organize microtubule minus ends into a pole structure. In contrast, our findings with ZYG-9 and TAC-1 suggest that ZYG-9 has a distinct, broader function in meiotic spindles that is not restricted to keeping acentrosomal poles focused and stable, perhaps through regulating the dynamics of microtubules in the center of the spindle. Teasing apart the contributions of different proteins to maintaining spindle stability is an important area of future research.

In summary, our degron-based experiments have provided new insight into the role of ZYG-9 in stabilizing the acentrosomal spindle and suggest that proper regulation of microtubule dynamics is essential to maintain spindle pole integrity. Intriguingly, the spindle instability observed in our live imaging of acute ZYG-9 depletion is reminiscent of the instability that has been observed in human oocytes [1]. Further experimentation from the same group recently revealed that spindle instability in human oocytes is connected to low levels of the kinesin-14 HSET [47]. ZYG-9 is now an additional factor that could be explored in mammalian oocytes. Moreover, applying degron-based depletions and FRAP experimentation to other pole proteins in *C*. *elegans* oocytes may help develop a more complete understanding of how oocyte meiotic spindles are stabilized, to ensure faithful segregation in the absence of centrosomes.

## Materials and methods

### *C*. *elegans* strains

A list of *C*. *elegans* strains used in this study is provided in Table 1.

**Table 1:** Strains used in this study.

Name	Description	Genotype
CA1199	*P_sun-1_::TIR1::mRuby*	*unc-119(ed3); ieSi38 [Psun-1*::*TIR1*::*mRuby*::*sun-1 3’UTR*, *cb-unc-119(+)] IV*
EU1067	*GFP*::*H2B* *GFP*::*tubulin*	*unc-119(ed3) ruIs32 [unc-119(+) pie-1*::*GFP*::*H2B] III; ruIs57 [unc-119(+) pie-1*::*GFP*::*tubulin]*
EU2861	*GFP*::*aspm-1*	*or1935 [GFP*::*aspm-1] I*
EU2876	*GFP*::*aspm-1* *mCherry*::*H2B*	*or1935 [GFP*::*aspm-1] I; ltIs37 [pAA64; pie-1*::*mCherry*::*his-58; unc-119(+)] IV*
JA1559	*mCherry*::*tubulin*	*weIs21 [pJA138 (pie-1*::*mCherry*::*tub*::*pie-1)]; unc-119(ed3) III*
OD56	*mCherry*::*H2B*	*ltIs37 [(pAA64) pie-1*::*mCherry*::*his-58 + unc-119(+)] IV*
OD57	*mCherry*::*H2B* *GFP*::*tubulin*	*unc-119(ed3) III; ltIs37 [pAA64; pie-1*::*mCherry*::*his-58; unc-119(+)] IV; ltIs25 [pAZ132; pie-1*::*GFP*::*tba-2; unc-119 (+)]*
PHX3422	*GFP*::*degron*::*tac-1* *P_sun-1_::TIR1::mRuby*	*tac-1(syb3422) II; ieSi38 IV*
SMW21	*P_sun-1_::TIR1::mRuby* *mCherry*::*H2B*	*unc-119(ed3); ieSi38 [Psun-1*::*TIR1*::*mRuby*::*sun-1 3’UTR*, *cb-unc-119(+)] IV; ltIs37 [(pAA64) pie-1*::*mCherry*::*his-58 + unc-119(+)] IV*
SMW22	*P_sun-1_::TIR1::mRuby* *mCherry*::*tubulin*	*unc-119(ed3) III; weIs21 [pJA138 (pie-1*::*mCherry*::*tubulin*::*pie-1)]; ieSi38 IV*
SMW24	*degron*::*GFP*::*zyg-9* *P_sun-1_::TIR1::mRuby*	*Pzyg-9*::*degron*::*EmGFP*::*zyg-9 (C6->T—PAM site mutation) II; unc-119(ed3); ieSi38 [Psun-1*::*TIR1*::*mRuby*::*sun-1 3’UTR*, *cb-unc-119(+)] IV*
SMW26	*degron*::*GFP*::*zyg-9* *mCherry*::*H2B* *P_sun-1_::TIR1::mRuby*	*Pzyg-9*::*degron*::*EmGFP*::*zyg-9 (C6->T—PAM site mutation) II; unc-119(ed3); ieSi38 [Psun-1*::*TIR1*::*mRuby*::*sun-1 3’UTR*, *cb-unc-119(+)] IV; ltIs37 [(pAA64) pie-1*::*mCherry*::*his-58 + unc-119(+)] IV*
SMW31	*P_sun-1_::TIR1::mRuby* *GFP*::*H2B* *GFP*::*tubulin*	*unc-119(ed3) III; ruIs32[unc-119(+) pie-1*::*gfp*::*h2b] III; ruIs57[unc-119(+) pie-1*::*GFP*::*tubulin]; ieSi38 IV*
SMW33	*degron*::*GFP*::*zyg-9* *mCherry*::*tubulin* *P_sun-1_::TIR1::mRuby*	*Pzyg-9*::*degron*::*EmGFP*::*zyg-9 (C6->T—PAM site mutation) II; unc-119(ed3); ieSi38 [Psun-1*::*TIR1*::*mRuby*::*sun-1 3’UTR*, *cb-unc-119(+)] IV; weIs21 [pJA138 (pie-1*::*mCherry*::*tub*::*pie-1)]; unc-119(ed3) III*
SMW71	*GFP*::*aspm-1* *degron*::*GFP*::*zyg-9* *mCherry*::*tubulin* *P_sun-1_::TIR1::mRuby*	*or1935 [GFP*::*aspm-1] I; Pzyg-9*::*degron*::*EmGFP*::*zyg-9 (C6->T—PAM site mutation) II; ieSi38 [Psun-1*::*TIR1*::*mRuby*::*sun-1 3’UTR*, *cb-unc-119(+)] IV; weIs21 [pJA138 (pie-1*::*mCherry*::*tub*::*pie-1)]; unc-119(ed3) III*

### Generation of degron::EmGFP::ZYG-9 strain (SMW24)

A CRISPR-based approach [48,49] was used to generate an endogenously tagged degron::EmGFP::ZYG-9 (SMW24). Briefly, 27μM recombinant Alt-R *S*. *pyogenes* Cas9 protein (IDT) was co-injected with 13.6μM tracrRNA (IDT), 4μM *dpy-10* crRNA, 1.34μM *dpy-10* repair oligo, 9.6μM *zyg-9* crRNA, and 136ng/μL ssDNA *zyg-9* repair template into CA1199 (Psun-1::TIR1::mRuby) worms, that were then allowed to produce progeny (See Table 2 for list of tracrRNA, crRNA, and primers used). Worms from plates containing rollers and dumpys were screened for degron::GFP insertions by PCR screening. To make the *zyg-9* repair template, we generated an N-terminal degron::EmGFP::linker (pADR28) using site directed mutagenesis and pLZ29 (gift from Abby Dernburg). The linker we inserted is from pIC26. The tag was then amplified using PCR with primers that contained homology to the *zyg-9* locus with the final product containing 57 bp of homology upstream of the *zyg-9* start codon and 61 bp of homology downstream of the start codon. ssDNA was generated by asymmetric PCR. SMW24 (degron::EmGFP::ZYG-9) was crossed with SMW21 (mCherry::histone; TIR1::mRuby) to generate SMW26: *Pzyg-9-16*::*degron*::*EmGFP*::*ZYG- 9 (C6->T—PAM site mutation) II; ieSi38 [Psun-1*::*TIR1*::*mRuby*::*sun-1 3’UTR*, *cb-unc- 119(+)] IV, ltIs37 [(pAA64) pie-1*::*mCherry*::*his-58 + unc-119(+)] IV*. SMW24 was also crossed with SMW22 to generate SMW33: *Pzyg-9*::*degron*::*EmGFP*::*zyg-9 (C6->T—PAM site mutation)* II; *unc-119(ed3)*; *ieSi38 [Psun-1*::*TIR1*::*mRuby*::*sun-1 3’UTR*, *cb-unc-119(+)] IV; weIs21 [pJA138 (Ppie-1*::*mCherry*::*tub*::*pie-1 3’UTR)]; unc-119(ed3) III*.

**Table 2:** CRISPR/Cas9 Information.

Alt-R CRISPR-Cas9 tracrRNA	Proprietary from IDT
*dpy-10* crRNA	5’-GCUACCAUAGGCACCACGAG-3’
*dpy-10* repair oligo (Ultramer from IDT)	5’-CACTTGAACTTCAATACGGCAAGATGAGAAT GACTGGAAACCGTACCGCATGCGGTGCCTATG GTAGCGGAGCTTCACATGGCTTCAGACCAACAGCCTAT-3’
*zyg-9* crRNA	5’-CUCGUCCAGAUAAUCCCAAU-3’
*zyg-9* repair-F1	5’-ACGTAGTAAACTGTCATTTTTCAGATAATGCCTAAA GATCCAGCCAAACC-3’
*zyg-9* repair-R1	5’-CCACCTCGTCCAGATAATCCCAATTAGACATTCTAG AGCGGCCGCCA-3’
*zyg-9* repair-F2	5’-TTCGTTCGCTTTCTTTGTTTATTGCAAGGCACGTA GTAAACTGTCATTTTTCAG-3’
*zyg-9* repair-R2	5’-CGAAGTTCGGTGGAAGTTTGGGAAGGATATCCACC TCGTCCAGATAATC-3’
*zyg-9* insertion check-F	5’-CGGAAATCTATTGTTGAAATCTCCTTTC-3’
*zyg-9* insertion check-R	5’-CTTTCATTTTTTCGAAAATGACGGG-3’
pADR28	degron::EmGFP::linker plasmid template for PCR reactions to generate *zyg-9* repair template. Derived from pLZ29.

### RNAi

RNAi was performed as in [50,51]. Briefly, from a feeding library [52,53], individual RNAi clones were picked and grown overnight at 37°C in LB with 100μg/ml ampicillin. Overnight cultures were spun down and plated on NGM (nematode growth media) plates containing 100μg/ml ampicillin and 1mM IPTG. Plates were dried overnight. Worm strains were synchronized by bleaching gravid adults and letting the eggs hatch overnight without food. L1s were then plated on RNAi plates and grown to adulthood at 15°C for 5–6 days.

### Immunofluorescence and antibodies

Immunofluorescence was performed as in [51]. Briefly, worms were dissected into M9 or Meiosis Medium (0.5 mg/mL Inulin from dahlia tubers (CAS Number 9005-80-5), 25 mM HEPES, pH 7.5, 60% Leibovitz’s L-15 Media (Gibco 11415–049), 20% Heat-inactivated Fetal Bovine Serum [54]), freeze cracking embryos, and plunging into -20°C methanol. Embryos were fixed for 35–45 minutes, rehydrated in PBS, and blocked in AbDil (PBS plus 4% BSA, 0.1% Triton X-100, 0.02% Na-Azide) for 30 minutes. Primary antibodies were incubated overnight at 4°C. The next day, embryos were washed 3x with PBST (PBS plus 0.1% Triton X-100), incubated in secondary antibody for 1 hour and 15 minutes, washed again as before, incubated in mouse anti-α-tubulin-FITC for 1.5 hours, washed again, and incubated in Hoechst (1:1000 in PBST) for 15 minutes. Embryos were then washed 2x with PBST, mounted in 0.5% p-phenylenediamine, 20mM Tris-Cl, pH 8.8, 90% glycerol, and sealed with nail polish; except for the overnight primary, the entire procedure was performed at room temperature. For experiments with staining of GFP::ZYG-9 (SMW24) using mouse anti-GFP, embryos were blocked in AbDil overnight at 4°C and incubated in primary antibody for 2 hours at room temperature. Primary antibodies used in this study: rabbit anti-ASPM-1 (1:5000, gift from Arshad Desai) [31], mouse anti-GFP (1:250; Invitrogen). Directly conjugated mouse anti-α-tubulin-FITC (DM1α, Sigma) and Alexa-fluor directly conjugated secondary antibodies (Invitrogen) were used at 1:500. All antibodies were diluted in AbDil.

### Generation of TAC-1 antibody

Based on previous predictions of TAC-1 domains and structure [14], we selected a 15aa sequence (PFNGSQNGHPENEEP) from the N-terminus of TAC-1. A rabbit polyclonal antibody was produced by ProteinTech, and the final antibody was affinity purified using a Sulfolink Immobilization Kit to generate the column. This antibody was validated for proper localization via IF imaging in both *control(RNAi)* and *tac-1(RNAi)* conditions. Due to relatively high background staining, this antibody was preabsorbed prior to use in immunofluorescence imaging. To preabsorb, ~100 worms (from the same background strain as the experimental slides) were grown under *tac-1(RNAi)* conditions. These worms were dissected at adulthood and subjected to our standard fixation methods. After blocking, these worms were incubated with TAC-1 antibody (1:50 diluted in AbDil) and left overnight at 4°C. The following day, the TAC-1 solution was removed and stored for 24–48 hours at 4°C prior to being used in another set of IF slides containing the experimental conditions.

### Embryonic lethality assays

For each strain, embryonic lethality and brood size was measured across 12 individual worms. Each worm was selected at the L4 stage and singled onto a NGM plate. Individual worms were moved from their plates every day to a fresh plate, and the previous plate was scored 24 hours later for the number of eggs and hatched worms. This process was carried out for 4 days of counting (until worms began to lay unfertilized oocytes due to lack of sperm) and values were pooled across all days for a single worm, then averaged across all worms. In the case of auxin treatment, the same process was repeated but with auxin-containing NGM plates. Results are shown in Table 3.

**Table 3:** Embryonic Lethality Assays of AID strains.

Strain	Auxin Treatment	Average Brood Size	Embryonic Lethality
**SMW24 (ZYG-9 AID)**	No	246 +/- 20	0.37% +/- 0.2%
	Yes	243 +/- 22	98.2% +/- 0.6%
**PHX3422 (TAC-1 AID)**	No	231 +/- 20	0.43% +/- 0.4%
	Yes	226 +/- 12	98.6% +/- 0.7%

### Microscopy

All fixed imaging was performed on a DeltaVision Core deconvolution microscope with a 100x objective (NA = 1.4) (Applied Precision). This microscope is housed in the Northwestern University Biological Imaging Facility supported by the NU Office for Research. Image stacks were obtained at 0.2μm z-steps and deconvolved using SoftWoRx (Applied Precision). All images in this study were deconvolved and displayed as full maximum intensity projections of data stacks encompassing the entire spindle structure, unless stated otherwise.

### Time-lapse imaging

Two-color live imaging was performed using a spinning disk confocal microscope with a 63x HC PL APO 1.40 NA objective lens. A spinning disk confocal unit (CSU-X1; Yokogawa Electric Corporation) attached to an inverted microscope (Leica DMI6000 SD) and a Spectral Applied Imaging laser merge ILE3030 and a back-thinned electron-multiplying charge-coupled device (EMCCD) camera (Photometrics Evolve 521 Delta) were used for image acquisition. The microscope and attached devices were controlled using Metamorph Image Series Environment software (Molecular Devices). The spinning disk microscope is housed in the Northwestern University Biological Imaging Facility supported by the NU Office for Research.

For *ex utero* imaging, adult worms were picked into a 10μL drop of Meiosis Medium in the center of a custom-made apparatus for live imaging [30,54]. All worms were quickly dissected, and an eyelash pick was used to push remaining worm bodies to the outside of the drop, leaving only the oocytes and embryos in the center to avoid disruption from worm movement during the imaging process. Vaseline was laid in a ring around the drop through a syringe, and a 18x18mm #1 coverslip was laid on top of the Vaseline ring, sealing the drop. This sealed slide was moved immediately to the Spinning Disk stage and inverted, allowing oocytes and embryos to float down to the surface of the coverslip and be subsequently imaged. Typically, ten to fifteen z-stacks at 1μm increments were taken every 15–30 seconds at room temperature. Images were processed using ImageJ. Images are shown as maximum intensity projections of the entire spindle structure.

Due to technical issues with the Leica Spinning Disk towards the end of this work, all GFP::ASPM-1 timelapses (Figs 2B and 6B) were acquired on a different microscope. These timelapses were collected using a Zeiss Axio Observer 7 inverted confocal microscope equipped with LSM800 GaAsP-PMT detectors and a Plan-Apochromat ×40 objective (Zeiss, 1.3 numerical aperture, oil immersion) and the supplied Zen Blue software (2.3 system). For acquisitions, 15 z-slices at 1μm increments were taken every 30 seconds at room temperature. Raw timelapses were processed via ImageJ; the image at each timepoint is a maximum intensity projection to display the entire spindle structure.

For experiments involving metaphase arrest, we did not image spindles that exhibited hallmarks of prolonged metaphase arrest when they were dissected (e.g., stretching of chromosomes and a disrupted spindle structure), to avoid artifacts arising from the metaphase-arrest condition.

### Auxin treatment

For long-term auxin treatment, worms were transferred onto NGM plates containing 1mM auxin (indol-3-acetic acid, Alfa Aesar) seeded with OP-50, incubated overnight, and then processed for immunofluorescence as described above.

For short-term auxin treatment for fixed imaging, whole worms were picked into a drop of Meiosis Medium and incubated for 25–30 minutes in a humidity chamber before dissection and freeze cracking. For vehicle treatment, 0.25% ethanol in Meiosis Medium was used. The rest of the protocol is the same as the immunofluorescence procedure described above.

For acute auxin treatment of oocytes for live imaging, worms were picked into a drop of Meiosis Medium containing either 100 μM auxin or vehicle (ethanol). The worms were quickly dissected to allow oocytes to enter the media, and mounted for imaging as described in the time lapse imaging section. For more details on auxin protocols, see [30,55].

### Western blotting

300 whole adult worms (per sample) were grown on control (empty vector) RNAi plates and treated in a manner similar to the auxin protocols utilized in this paper (30 minutes in auxin solution for short-term AID, 240 minutes on auxin plates for long-term AID). Worms were subsequently bleached to remove worm bodies and harvest embryos, and these samples were spun down (800 rcf for 1 minute), rinsed in M9 and spun down again, and then M9 was removed to leave only clean embryos. The pellet was then mixed with SDS lysis buffer, boiled for 10 minutes (using a heat block at 95°C), and run on a 4–20% gradient Tris-Glycine gel (BioRad Mini-PROTEAN TGX) at 80-100V for ~1.5 hours. Protein was transferred onto nitrocellulose via a BioRad Trans-blot Turbo apparatus (semi-dry in a 10% MeOH, 25mM Tris, 192mM glycine transfer buffer) at 25V for 30 minutes. Blot was placed on rocker and blocked in 5% milk in TBS/0.1%Tween overnight at 4°C, separated into two pieces, and then incubated with indicated primary antibodies overnight at 4°C (1:1000 mouse anti-degron or 1:5000 mouse anti-tubulin). The entire blot was then washed with TBS/0.1% Tween, treated with anti-mouse HRP (1:5000) for 1.5 hours at RT, washed again, incubated for 4 minutes in BioRad Clarity ECL solution, and then exposed for 0.5 to 5 minutes, depending on brightness of bands.

### FRAP

FRAP experiments were performed on the same spinning disk confocal microscope mentioned above using a 63x HC PL APO 1.40 NA oil immersion objective. Photobleaching was performed with a 405 nm laser (5.5 mW-11.0 mW output). Poles were bleached using 5 repetitions of 100ms and images were taken at 5-10s intervals. Analysis of the recovery curves and the half-time recovery were carried out with FIJI to obtain raw fluorescence data and the curves were fit using a custom Python script (https://github.com/justinfinkle/mullen-frap). For all FRAP acquisitions except ASPM-1 meiotic spindles, oocytes and embryos were mounted as described in the time lapse imaging section. Bleaching of the mitotic centrosomes was done in the EMS cell in the 4-cell embryo, and MI and MII meiotic spindles were bleached for the acentrosomal spindle pole FRAP experiments. For ASPM-1 meiotic spindle FRAP experiments live, intact worms were mounted on 3–5% agarose, M9 pads in 50% live imaging solution (modified S-basal [50mM KH2PO4,10mM K-citrate, 0.1M NaCl, 0.025mg/ml cholesterol, 3mM MgSO4, 3mM CaCl2, 40mM serotonin creatinine sulfate monohydrate]), 50% 0.1 micron polystyrene Microspheres (Polysciences Inc.), and covered with a coverslip. Only data from bleached spindles that progressed to anaphase were included in our analysis.

### Data analysis and quantification

**Fig 1B:** A 10 pixel wide x 12μm line profile analysis was performed in ImageJ on max projected images of 10 different metaphase spindles after background subtraction.

**Fig 1C:** IF images of oocyte spindles were used for linescan measurements of ASPM-1 and ZYG-9 channels. A 3 x 12μm line profile analysis was performed in ImageJ on max projected images of 23 different metaphase spindles after background subtraction.

**Fig 1D:** IF images of oocyte spindles stained for DNA, tubulin, ASPM-1, and ZYG-9 were scored as bipolar if ASPM-1 was enriched at two poles, or multipolar/collapsed if ASPM-1 localized to multiple foci or was diffuse throughout the spindle. In oocytes from worms not treated with auxin, 1/34 spindles from 2 biological replicates were multipolar. In oocytes from worms plated on auxin-containing plates for 24 hours, 44/45 spindles from 2 biological replicates were multipolar or collapsed.

**Fig 1E:** IF images of 1-cell stage embryos stained for DNA, tubulin, ASPM-1, and ZYG-9 were scored as aligned if the spindle was oriented parallel to the A-P axis of the embryo, or misaligned if the spindle was oriented perpendicular to this axis. In zygotes from worms not treated with auxin, 4/4 spindles from 2 biological replicates were aligned. In zygotes from worms plated on auxin-containing plates for 24 hours, 8/8 spindles from 2 biological replicates were misaligned.

**Fig 3C:** Imaris 3D Imaging Software (Bitplane) was used for spindle length measurements (procedure modified from [56,57]). To calculate spindle length, the “Surfaces” tool was first used to determine the volume of each pole stained with ASPM-1 and then to assign the center of the volume for each pole. The distance between these two center points was then measured as the spindle length.

**Fig 3D:** Pole phenotypes were quantified by eye in unarrested *(control(RNAi))* and Metaphase I-arrested *(emb-30(RNAi))* oocytes in the presence of either vehicle or 1 mM auxin. Spindle poles were scored as fragmented if there were clear defects in spindle pole organization including excess tubulin and ASPM-1 signal in the area near the spindle poles and splaying or fragmentation of the tubulin and ASPM-1 signal at the poles. In unarrested conditions without auxin, 99/106 (93.4%) poles were focused while 7/106 (6.6%) were fragmented. With addition of auxin, we observed 13/79 (16.5%) poles were focused while 66/79 (83.5%) were fragmented. For metaphase-arrested conditions without auxin, 81/86 (94.2%) poles were focused while 5/86 (5.8%) were fragmented. With addition of auxin, we observed 27/90 (30%) poles were focused while 63/90 (70%) were fragmented.

**Fig 3E:** Midspindle bundles were quantified using the Segmented Line tool within FIJI. Based on the average width of a visible bundle, a linescan was manually fit to the curvature of a single bundle that ran laterally across a chromosome at the metaphase plate (4 μm long by 0.25 μm wide); each linescan was visually confirmed through the DNA channel to be centered based on the corresponding chromosome and to be aligned with it. Using a sum-projected image of the tubulin channel, the average intensity across the bundle was measured. In addition, the positions of the start/end of the chromosome in relation to the linescan were recorded to accurately represent the chromosome length in visualization of the data. Finally, to account for background fluorescence and normalize all spindles, a 50x50 pixel ROI was drawn within the cytoplasm of the oocyte to acquire a mean background intensity for every stack measured. All linescans were averaged together to generate a mean intensity value across the entire length, and all chromosome lengths were averaged to generate the gray shaded area that represents the location of the chromosome in relation to the linescan. SEM was calculated, and +/- SEM was applied to the mean value to generate final shaded areas on graph.

**Fig 5B:** Oocyte spindles were quantified using the FIJI plugin Coloc2 to determine the Pearson coefficient between the ZYG-9 and ASPM-1 channels. All r values were placed into boxplots using Rstudio (boxes represent first quartile, median, and third quartile); the mean values of the Pearson coefficient in *control(RNAi)* and *tac-1(RNAi)* conditions were compared against each other using a two-tailed t-test. Images for each condition were collected from at least three biological replicates, and the number of images for each condition was listed above the boxplots.

**Fig 5D:** Spindle length was measured as described for Fig 3C.

**Fig 5E:** Pole phenotypes were quantified by eye as described for Fig 3D. In unarrested conditions without auxin, 35/38 (92.1%) poles were focused while 3/38 (7.9%) were fragmented. With addition of auxin, we observed 7/58 (12.1%) poles were focused while 51/58 (87.9%) were fragmented. For metaphase-arrested conditions without auxin, 28/30 (93.3%) poles were focused while 2/30 (6.7%) were fragmented. With addition of auxin, we observed 4/51 (7.8%) poles were focused while 47/51 (92.2%) were fragmented.

**Fig 5F:** Midspindle bundles were quantified using the Segmented Line tool within FIJI, using the method described for Fig 3E.

**Fig 6A:** Representative movies of embryos dissected into either control Meiosis Medium or medium containing auxin (to deplete ZYG-9) are shown. Following auxin treatment, monopolar spindle organization was not disrupted upon ZYG-9 depletion in 5/5 movies.

**Fig 6D and 6F:** The volume of the spindle pole was determined using ASPM-1 staining as described in [58]. Briefly, Imaris 3D Imaging Software (Bitplane) was used for spindle pole volume measurements. The “Surfaces” tool was used to determine the volume of each pole, using ASPM-1 staining to define the pole. Images for each condition were collected from at least three biological replicates.

**S1B Fig:** For panel B, we captured multiple images of each stage, which all showed the localization pattern displayed in the representative images. The number of images captured were: 6 Pre-NEBD, 11 Cage/Multipolar, 9 Multipolar, 41 Bipolar, 16 Early anaphase, and 14 Late anaphase.

**S2 Fig:** Verification of antibody staining was done by comparing *control(RNAi)* and *tac-1(RNAi)* worms. Localization of TAC-1 and ZYG-9 to centrosomes was consistent across 5/5 *control(RNAi)* embryos and loss of TAC-1 and ZYG-9 at centrosomes was observed in 5/5 *tac-1(RNAi)* embryos. For oocytes, TAC-1 and ZYG-9 localization to acentrosomal poles was observed in 5/5 *control(RNAi)* oocytes and loss of TAC-1 and ZYG-9 localization to acentrosomal poles was observed in 5/5 *tac-1(RNAi)* oocytes.

**S3B Fig:** Pole phenotypes were quantified by eye as described for Fig 3D. Exact percentages for *control(RNAi)* were 95.1% (78/82) focused, 4.9% (4/82) fragmented (No Auxin), and 18.1% (17/94) focused, 81.9% (77/94) fragmented (With Auxin). Exact percentages for *tac-1(RNAi)* were 14.1% (9/64) focused, 85.9% (55/64) fragmented (No Auxin) and 11.8% (6/51) focused, 88.2% (45/51) fragmented (With Auxin). Images for each condition were collected from at least three biological replicates.

**S3C Fig:** Midspindle phenotypes were quantified by eye in unarrested *(control(RNAi))* and Metaphase-I arrested *(emb-30(RNAi))* oocytes in the presence of either vehicle or 1 mM auxin. Midspindle microtubule splaying was determined by observing microtubule bundles near chromosomes. If microtubules could be seen splaying outwards into the cytoplasm, with no clear connection to another microtubule bundle or to the chromosomes, that spindle was considered splayed. Exact percentages for *control(RNAi)* were 91.4% (75/82) bundled, 8.6% (7/82) splayed (No Auxin) and 22.3% (21/94) bundled, 77.7% (73/94) splayed (With Auxin). Exact percentages for *tac-1(RNAi)* were 31.3% (20/64) bundled, 68.7% (44/64) splayed (No Auxin) and 27.5% (14/51) bundled, 72.5% (37/51) splayed (With Auxin). Images for each condition were collected from at least three biological replicates.

### Statistical methods

All statistical analysis was done using a student’s two-tailed t test. Data distributions were confirmed to be normal via the Shapiro-Wilk Test.

## Supporting information

S1 Fig Additional validation of the ZYG-9 AID strain. (A) Movie stills from an oocyte expressing degron::GFP::ZYG-9 (green) and mCherry::tubulin (magenta). ZYG-9 initially localizes to the spindle microtubules at the multipolar stage (6:00) and becomes progressively enriched on the spindle poles as meiosis progresses (9:00–19:00) before dissociating during anaphase (24:00). Bar = 5μm. Timestamp = min:sec. (B) Oocyte spindles stained for DNA (blue), tubulin (green), ZYG-9 (stained with a GFP antibody; red in merge), and ASPM-1. ZYG-9 localizes to spindle microtubules at the multipolar stage, becomes enriched at the spindle poles as spindle assembly proceeds, then begins to lose enrichment at the poles during anaphase. Bars = 2.5μm. **(C)** An embryo-only western blot demonstrating the effectiveness of both short-term and long-term AID depletion of ZYG-9 in the ZYG-9 AID strain expressing degron::GFP::ZYG-9 (predicted to be ~188 kDa). A faint band of ZYG-9 is present in control sample (middle lane), while no band is present in embryos following either short-term AID (30 minute treatment via soaking; left lane) or long-term AID (240 minute treatment on plates; right lane), validating the efficiency of AID depletion. Tubulin was used as a loading control and ZYG-9 was detected with an anti-degron antibody.
(TIF)

S2 Fig TAC-1 Antibody Validation. (A) Verification of TAC-1 antibody using IF imaging of one-cell mitotic embryos. TAC-1 localizes to centrosomes, and colocalizes with ZYG-9, as previously described [14,33]. No staining occurs following *tac-1(RNAi)*, demonstrating that TAC-1 staining is specific. Consistent with previous studies, TAC-1 depletion leads to loss of ZYG-9 at centrosomes and results in defects in mitotic spindle positioning and spindle length. Bars = 5μm. (B) Verification of TAC-1 antibody using IF imaging of oocyte spindles. TAC-1 is localized to acentrosomal poles and is colocalized with ZYG-9. Oocytes treated with *tac-1(RNAi)* do not have any TAC-1 signal and also lose localization of ZYG-9 to acentrosomal poles (5/5 spindles). Bars = 2.5μm.
(TIF)

S3 Fig Concurrent depletion of ZYG-9 and TAC-1 displays no difference in phenotypes when compared to ZYG-9 depletion alone. (A) IF imaging of oocyte spindles in either control or *tac-1(RNAi)* conditions. Shown are DNA (blue), tubulin (green), ASPM-1 (red), and ZYG-9 (stained with a GFP antibody; not shown in merge). Whether subjected to *tac-1(RNAi)* alone or concurrently with short-term ZYG-9 AID depletion, spindle phenotypes mimic those observed in short-term ZYG-9 AID depletion alone (zooms of poles and midspindle region in columns 6–9). Splaying highlighted with arrowheads. Bars = 2.5μm. (B, C) Quantification of acentrosomal pole fragmentation (B) or midspindle microtubule bundle splaying (C) from oocyte spindles observed in (A); total spindles counted in each condition are noted above stacked bars.
(TIF)

S4 Fig Validation of a TAC-1 AID strain. (A) IF imaging of embryos from worms grown on either control or auxin-containing plates. Shown are DNA (blue), tubulin (green), ASPM-1 (red), and TAC-1 (stained with a GFP antibody; not shown in merge). Embryos from TAC-1 AID worms treated with auxin display canonical phenotypes associated with *tac-1(RNAi)*, such as short astral microtubules and spindle positioning defects. Defects were consistent across 14/14 embryos. Bars = 2.5μm. (B) IF imaging of oocyte spindles from TAC-1 AID worms grown on either control or auxin-containing plates. Shown are DNA (blue), tubulin (green), ASPM-1 (red), and TAC-1 (stained with a GFP antibody; not shown in merge). Oocytes from worms treated with auxin display canonical phenotypes associated with *tac-1(RNAi)*, such as pole fragmentation and multipolar spindles. Defects in midspindle microtubule bundles were also prevalent across all spindles. Bars = 2.5μm. (C) An embryo-only western blot demonstrating the effectiveness of both short-term and long-term AID depletion of GFP::degron::TAC-1 (predicted to be ~60 kDa). A clear band of TAC-1 is present in control sample (left lane), while no band is visible following either short-term or long-term AID (middle and right lanes), validating the efficiency of AID depletion. α-tubulin was utilized as a loading control and TAC-1 was detected with an anti-degron antibody.
(TIF)

S5 Fig FRAP analysis with fit curves. (A-C) graphs of recovery curves and fit curves from the bleached poles of the FRAP experiments described in Fig 7. The mean is a solid line, the standard error of the mean is the shaded region, and the fit from the single exponential function is a dashed line. The t_1/2_’s were calculated from these fit curves.
(TIF)

S1 Video ZYG-9 localization during oocyte meiosis. *In utero* imaging of control oocyte expressing mCherry::tubulin and GFP::ZYG-9 (left); GFP::ZYG-9 alone is shown on the right. Corresponds to S1A Fig. ZYG-9 localizes to spindles at the multipolar stage, and is enriched at poles in metaphase and anaphase. Time elapsed shown in (min):(sec). Scale bar = 10μm.
(MOV)

S2 Video Auxin treatment rapidly depletes ZYG-9 and causes spindle instability. Shows a metaphase-arrested oocyte expressing mCherry::tubulin (left), dissected into Meiosis medium containing 100μM auxin to deplete ZYG-9. Corresponds to Fig 2A. Once dissected into auxin solution, rapid depletion of degron::GFP::ZYG-9 (right) is evident and spindle poles become unstable. Time elapsed shown in (min):(sec). Scale bar = 5μm.
(MOV)

S3 Video Auxin treatment rapidly depletes ZYG-9 and causes spindle instability. Another example of a metaphase-arrested oocyte expressing mCherry::tubulin (top), dissected into Meiosis medium containing 100μM auxin to deplete ZYG-9. Once dissected into auxin solution, rapid depletion of degron::GFP::ZYG-9 (bottom) is evident and spindle poles become unstable. Time elapsed shown in (min):(sec). Scale bar = 5μm.
(MOV)

S4 Video Metaphase-arrested spindles remain stable without auxin treatment. Shows a metaphase-arrested oocyte expressing mCherry::tubulin (left) and degron::GFP::ZYG-9 (right), dissected into a control Meiosis medium solution. Corresponds to Fig 2A. No major changes in spindle length or shape occur. Note that the spindle rotates end-on for a portion of the video, but when it rotates back it is clear that the morphology of spindle has not changed. Time elapsed shown in (min):(sec). Scale bar = 5μm.
(MOV)

S5 Video Timelapse of stable bipolar spindle expressing GFP::ASPM-1 and mCherry::tubulin. Shows a metaphase-arrested oocyte expressing degron::GFP::ZYG-9, GFP::ASPM-1, and mCherry::tubulin. On the left is a merge of ASPM-1 (green) and tubulin (magenta), and on the right is ASPM-1 alone (gray). When dissected into a control Meiosis Media solution, no major changes in spindle morphology were observed. Corresponds to Fig 2B. For the duration of the timelapse, ASPM-1 foci remained focused and stable at either end of the spindle. A faint population of GFP::ZYG-9 can be observed on spindle microtubules. Time elapsed shown in (min):(sec). Scale bar = 5μm.
(MOV)

S6 Video ASPM-1 marked poles split and fragment following acute ZYG-9 AID depletion. An example of a metaphase-arrested oocyte expressing degron::GFP::ZYG-9, GFP::ASPM-1, and mCherry::tubulin. On the left is a merge of ASPM-1 (green) and tubulin (magenta), and on the right is ASPM-1 alone (gray). When dissected into a Meiosis medium solution containing 100μM auxin, splitting and fragmentation of ASPM-1 foci manifested rapidly. Corresponds to Fig 2B. Disruption of stable acentrosomal lead to formation of a multipolar spindle and spindle collapse. Time elapsed shown in (min):(sec). Scale bar = 5μm.
(MOV)

S7 Video ASPM-1 marked poles split and fragment following acute ZYG-9 AID depletion. Another example of a metaphase-arrested oocyte expressing degron::GFP::ZYG-9, GFP::ASPM-1, and mCherry::tubulin. On the left is a merge of ASPM-1 (green) and tubulin (magenta), and on the right is ASPM-1 alone (gray). When dissected into a Meiosis medium solution containing 100μM auxin, splitting and fragmentation of ASPM-1 foci manifested rapidly. Corresponds to Fig 2B. Disruption of stable acentrosomal lead to formation of a multipolar spindle and spindle collapse. Time elapsed shown in (min):(sec). Scale bar = 5μm.
(MOV)

S8 Video Monopolar spindle organization does not change following acute ZYG-9 depletion. Shows a metaphase-arrested *klp-18(RNAi)* oocyte expressing mCherry::tubulin (left) and degron::GFP::ZYG-9 (right) dissected into Meiosis medium containing 100μM auxin to deplete ZYG-9. Corresponds to Fig 6A. Upon dissection into auxin solution, ZYG-9 is rapidly depleted but the organization of the monopole does not noticeably change. Time elapsed shown in (min):(sec). Scale bar = 5μm.
(MOV)

S9 Video Monopolar spindles remain stable without auxin treatment. Shows a metaphase-arrested *klp-18(RNAi)* oocyte expressing mCherry::tubulin (left) and degron::GFP::ZYG-9 (right) dissected into a control Meiosis Medium solution. Corresponds to Fig 6A. No major changes in monopole organization occurs during the course of the movie. Time elapsed shown in (min):(sec). Scale bar = 5μm.
(MOV)

S10 Video Timelapse of stable monopolar spindle expressing GFP::ASPM-1 and mCherry::tubulin. Shows a metaphase-arrested *klp-18(RNAi)* oocyte expressing GFP::ASPM-1, mCherry::tubulin (left) and degron::GFP::ZYG-9 (right) dissected into a control Meiosis Medium solution. Corresponds to Fig 6B. No major changes in monopole organization occurs during the course of the timelapse, visualized by a single ASPM-1 foci. Time elapsed shown in (min):(sec). Scale bar = 5μm.
(MOV)

S11 Video ASPM-1 marked monopole is not disrupted upon acute ZYG-9 AID depletion. Shows a metaphase-arrested *klp-18(RNAi)* oocyte expressing GFP::ASPM-1, mCherry::tubulin (left) and degron::GFP::ZYG-9 (right) dissected into a Meiosis Medium solution containing 100μM auxin. Corresponds to Fig 6B. Although ZYG-9 was depleted from the oocyte, no disruption of monopole organization could be observed. Time elapsed shown in (min):(sec). Scale bar = 5μm.
(MOV)

S12 Video FRAP of ZYG-9 at the poles of acentrosomal oocyte spindles. Shows a Metaphase II oocyte spindle labeled with degron::GFP::ZYG-9 and mCherry::histone; the polar body is on the left side of the spindle. During the video the pole on the left is photobleached but fluorescence quickly recovers. Corresponds to Fig 7A. Time elapsed shown in seconds. Scale bar = 2.5μm.
(MOV)

S13 Video FRAP of ZYG-9 at the poles of mitotic centrosome-containing spindles. Shows a mitotic spindle in the EMS cell of the 4-cell embryo, labeled with degron::GFP::ZYG-9 and mCherry::histone. During the video the pole on the right is photobleached. Fluorescence recovers but more slowly than on acentrosomal poles. Corresponds to Fig 7A. Time elapsed shown in seconds. Scale bar = 5μm.
(MOV)

S14 Video FRAP of tubulin at the poles of acentrosomal oocyte spindles. Shows a Metaphase II oocyte spindle labeled with degron::GFP::ZYG-9 and mCherry::histone; the polar body is on the right side of the spindle. During the video the pole on the bottom is photobleached but fluorescence quickly recovers. Corresponds to Fig 7B. Time elapsed shown in seconds. Scale bar = 2.5μm.
(MOV)

S15 Video FRAP of ASPM-1 at the poles of acentrosomal oocyte spindles. Shows a Metaphase II oocyte spindle labeled with GFP::ASPM-1 and mCherry::histone; the polar body is not visible in the selected z-stacks. During the video the pole on the left is photobleached. Corresponds to Fig 7C. Time elapsed shown in seconds. Scale bar = 2.5μm.
(MOV)

S16 Video FRAP of ASPM-1 at the poles of mitotic centrosome-containing spindles. Shows a mitotic spindle in the EMS cell of the 4-cell embryo, labeled with GFP::ASPM-1 and mCherry::histone. During the video the pole on the left is photobleached. Corresponds to Fig 7C. Time elapsed shown in seconds. Scale bar = 5μm.
(MOV)
