# Supplementary figures and images for: ZYG-9ch-TOG promotes the stability of acentrosomal poles via regulation of spindle microtubules in C. elegans oocyte meiosis
Source: PLoS Genet. 2022 Nov 30;18(11):e1010489. doi: 10.1371/journal.pgen.1010489 (PMC9757581; doi:10.1371/journal.pgen.1010489)

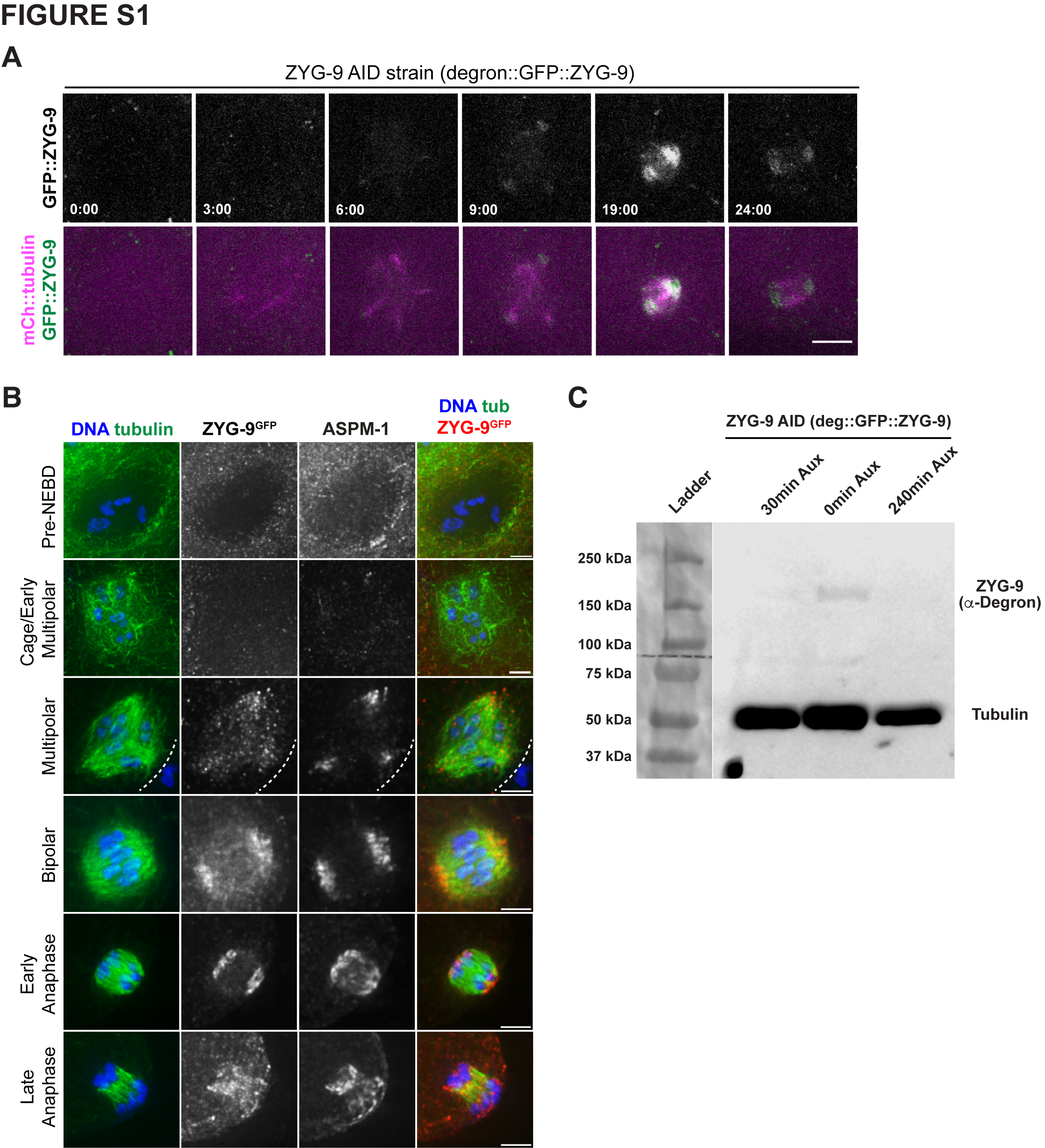

Supplement: S1 Fig — (A) Movie stills from an oocyte expressing degron::GFP::ZYG-9 (green) and mCherry::tubulin (magenta). ZYG-9 initially localizes to the spindle microtubules at the multipolar stage (6:00) and becomes progressively enriched on the spindle poles as meiosis progresses (9:00–19:00) before dissociating during anaphase (24:00). Bar = 5μm. Timestamp = min:sec. (B) Oocyte spindles stained for DNA (blue), tubulin (green), ZYG-9 (stained with a GFP antibody; red in merge), and ASPM-1. ZYG-9 localizes to spindle microtubules at the multipolar stage, becomes enriched at the spindle poles as spindle assembly proceeds, then begins to lose enrichment at the poles during anaphase. Bars = 2.5μm. (C) An embryo-only western blot demonstrating the effectiveness of both short-term and long-term AID depletion of ZYG-9 in the ZYG-9 AID strain expressing degron::GFP::ZYG-9 (predicted to be ~188 kDa). A faint band of ZYG-9 is present in control sample (middle lane), while no band is present in embryos following either short-term AID (30 minute treatment via soaking; left lane) or long-term AID (240 minute treatment on plates; right lane), validating the efficiency of AID depletion. Tubulin was used as a loading control and ZYG-9 was detected with an anti-degron antibody. (TIF) [file pgen.1010489.s001.tif]

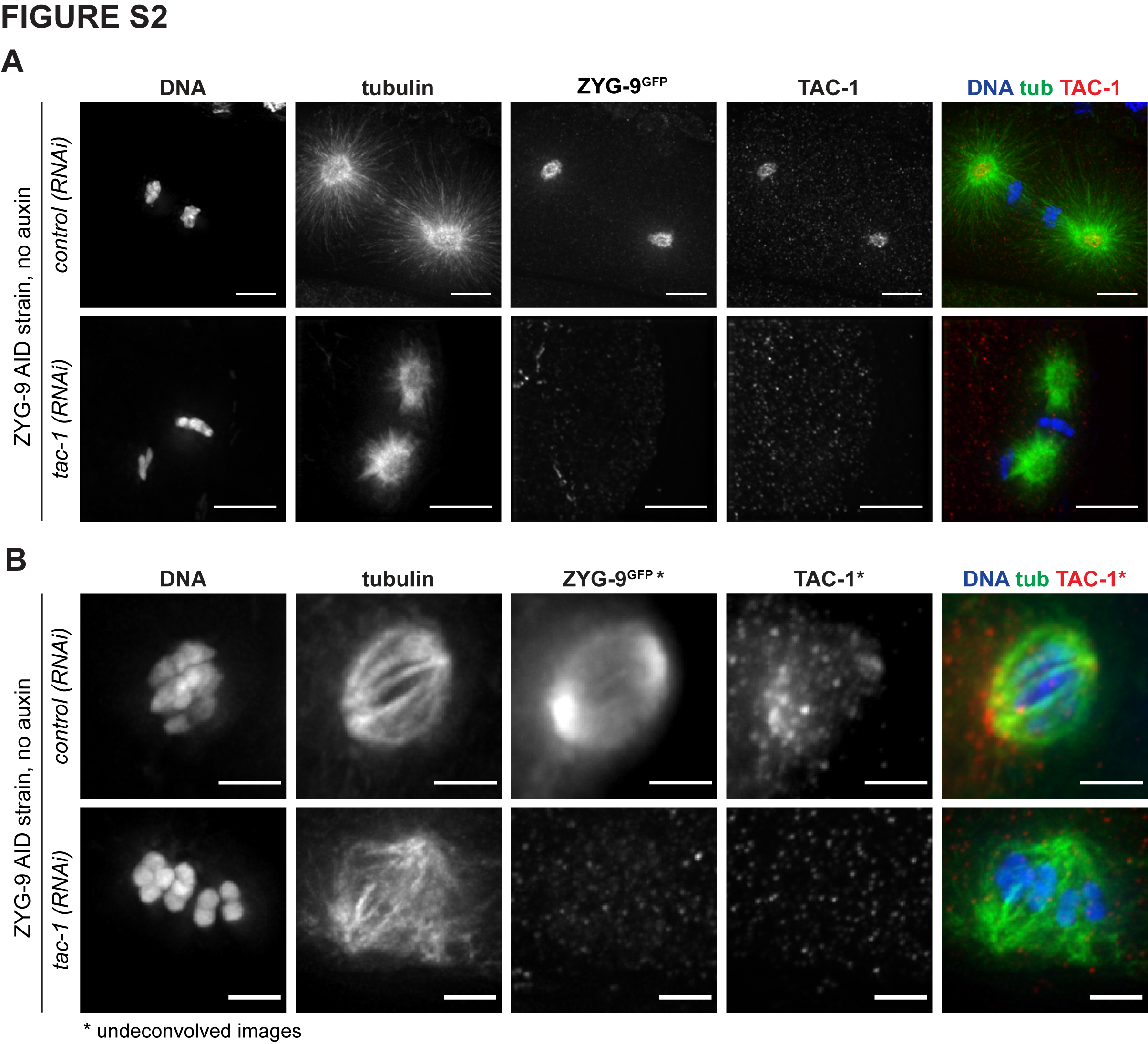

Supplement: S2 Fig — (A) Verification of TAC-1 antibody using IF imaging of one-cell mitotic embryos. TAC-1 localizes to centrosomes, and colocalizes with ZYG-9, as previously described [14,33]. No staining occurs following tac-1(RNAi), demonstrating that TAC-1 staining is specific. Consistent with previous studies, TAC-1 depletion leads to loss of ZYG-9 at centrosomes and results in defects in mitotic spindle positioning and spindle length. Bars = 5μm. (B) Verification of TAC-1 antibody using IF imaging of oocyte spindles. TAC-1 is localized to acentrosomal poles and is colocalized with ZYG-9. Oocytes treated with tac-1(RNAi) do not have any TAC-1 signal and also lose localization of ZYG-9 to acentrosomal poles (5/5 spindles). Bars = 2.5μm. (TIF) [file pgen.1010489.s002.tif]

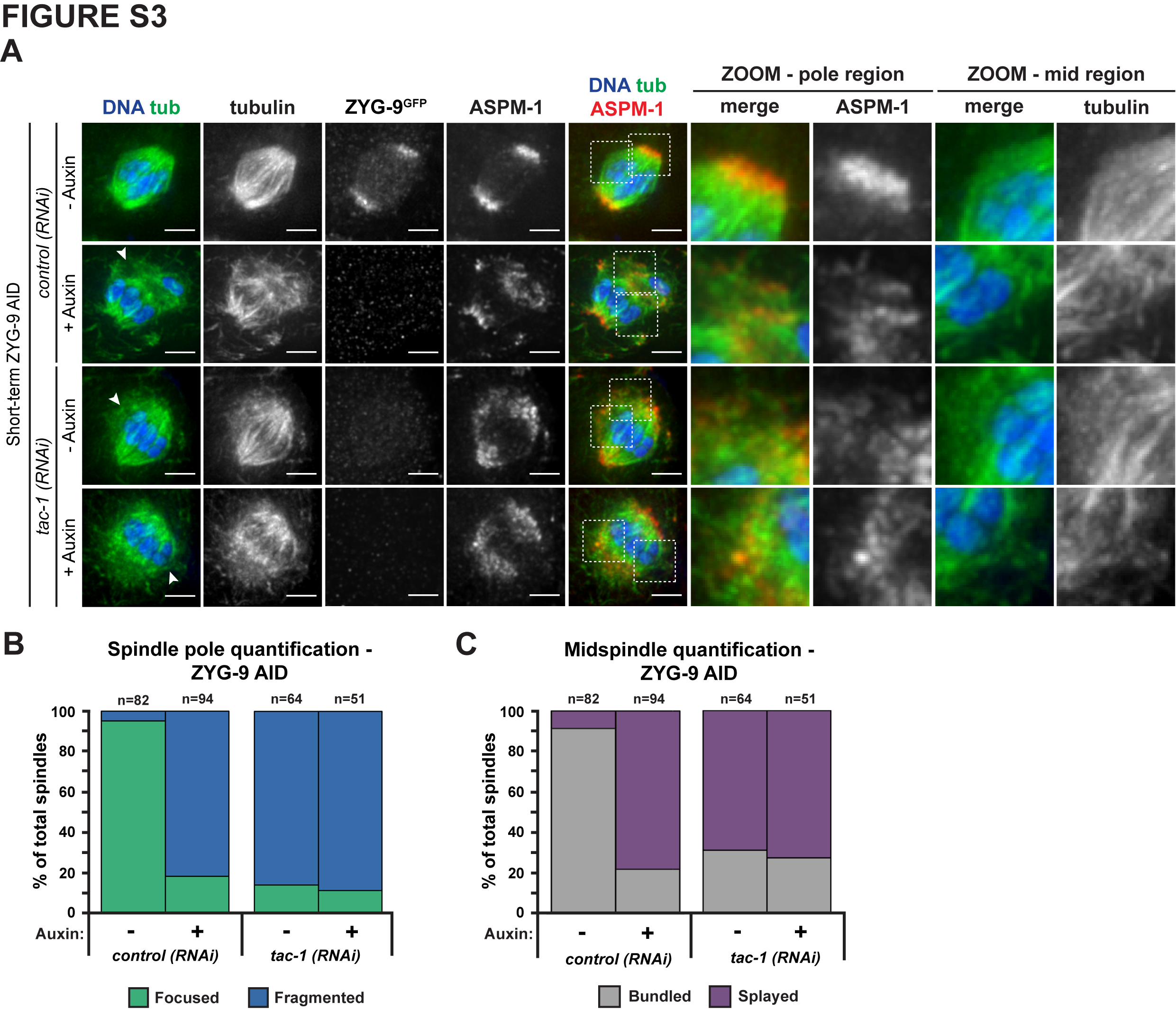

Supplement: S3 Fig — (A) IF imaging of oocyte spindles in either control or tac-1(RNAi) conditions. Shown are DNA (blue), tubulin (green), ASPM-1 (red), and ZYG-9 (stained with a GFP antibody; not shown in merge). Whether subjected to tac-1(RNAi) alone or concurrently with short-term ZYG-9 AID depletion, spindle phenotypes mimic those observed in short-term ZYG-9 AID depletion alone (zooms of poles and midspindle region in columns 6–9). Splaying highlighted with arrowheads. Bars = 2.5μm. (B, C) Quantification of acentrosomal pole fragmentation (B) or midspindle microtubule bundle splaying (C) from oocyte spindles observed in (A); total spindles counted in each condition are noted above stacked bars. (TIF) [file pgen.1010489.s003.tif]

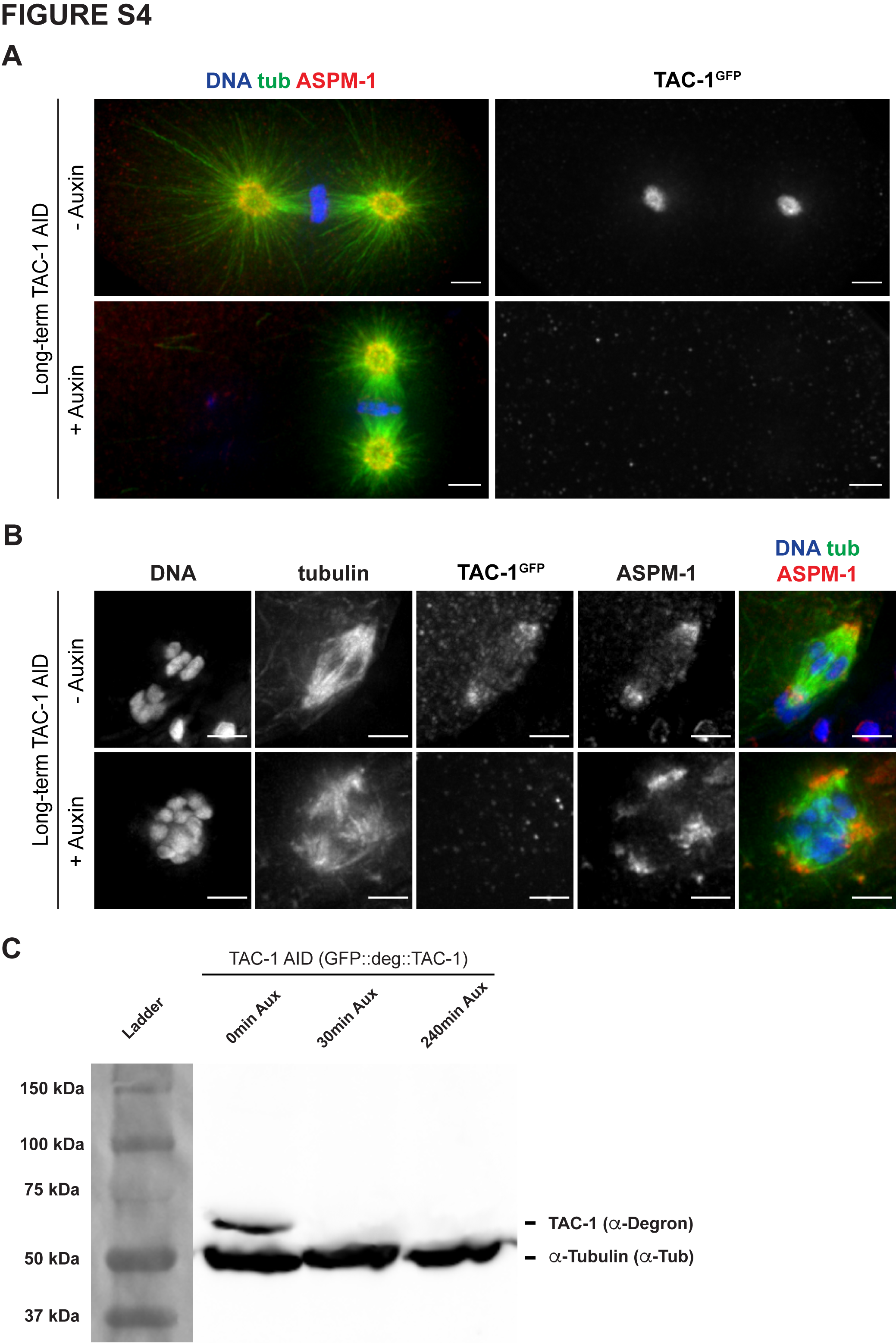

Supplement: S4 Fig — (A) IF imaging of embryos from worms grown on either control or auxin-containing plates. Shown are DNA (blue), tubulin (green), ASPM-1 (red), and TAC-1 (stained with a GFP antibody; not shown in merge). Embryos from TAC-1 AID worms treated with auxin display canonical phenotypes associated with tac-1(RNAi), such as short astral microtubules and spindle positioning defects. Defects were consistent across 14/14 embryos. Bars = 2.5μm. (B) IF imaging of oocyte spindles from TAC-1 AID worms grown on either control or auxin-containing plates. Shown are DNA (blue), tubulin (green), ASPM-1 (red), and TAC-1 (stained with a GFP antibody; not shown in merge). Oocytes from worms treated with auxin display canonical phenotypes associated with tac-1(RNAi), such as pole fragmentation and multipolar spindles. Defects in midspindle microtubule bundles were also prevalent across all spindles. Bars = 2.5μm. (C) An embryo-only western blot demonstrating the effectiveness of both short-term and long-term AID depletion of GFP::degron::TAC-1 (predicted to be ~60 kDa). A clear band of TAC-1 is present in control sample (left lane), while no band is visible following either short-term or long-term AID (middle and right lanes), validating the efficiency of AID depletion. α-tubulin was utilized as a loading control and TAC-1 was detected with an anti-degron antibody. (TIF) [file pgen.1010489.s004.tif]

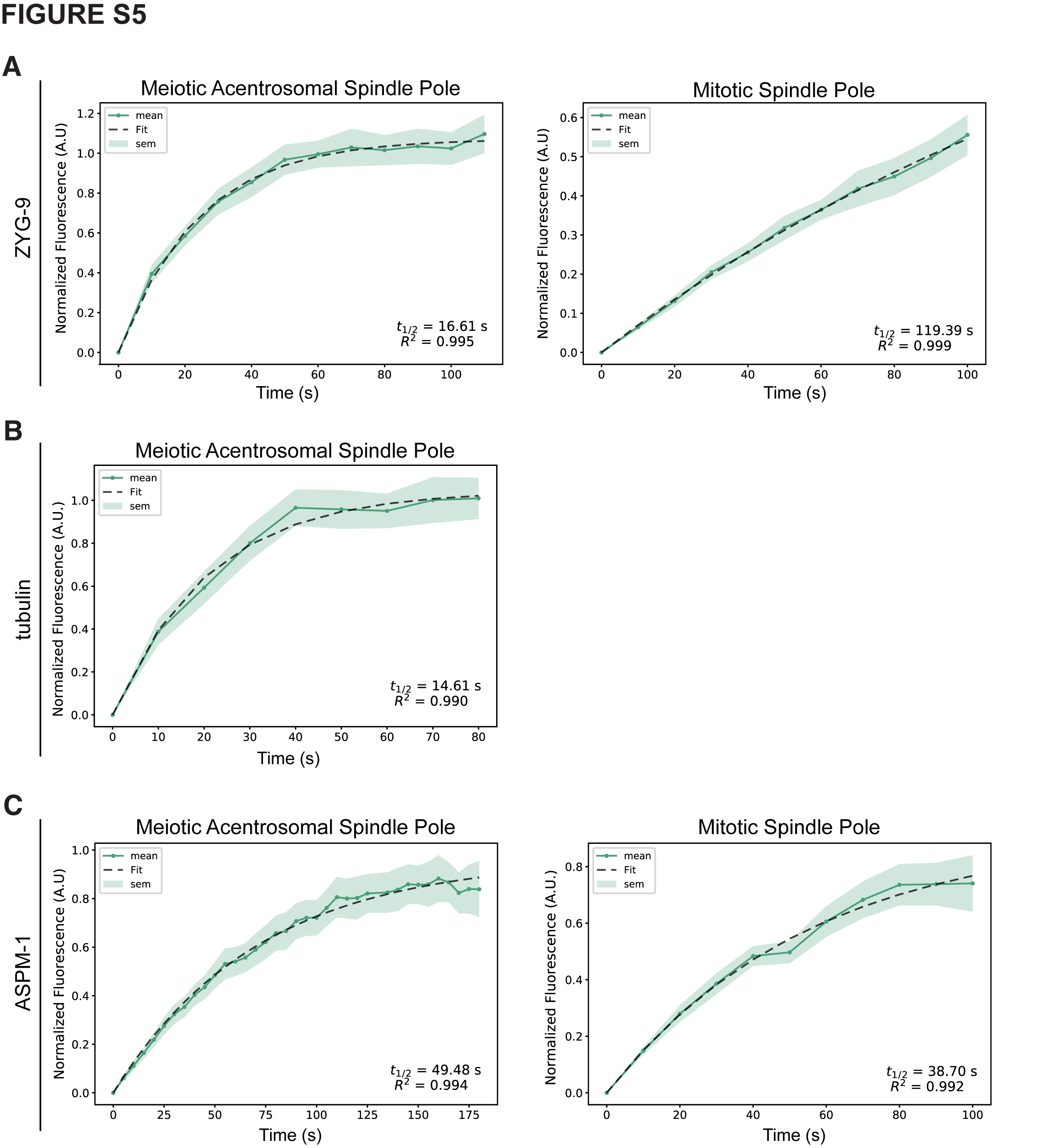

Supplement: S5 Fig — (A-C) graphs of recovery curves and fit curves from the bleached poles of the FRAP experiments described in Fig 7. The mean is a solid line, the standard error of the mean is the shaded region, and the fit from the single exponential function is a dashed line. The t1/2’s were calculated from these fit curves. (TIF) [file pgen.1010489.s005.tif]
